# Antisense ribosomal siRNAs inhibit RNA polymerase I-directed transcription in *C. elegans*

**DOI:** 10.1093/nar/gkab662

**Published:** 2021-08-07

**Authors:** Shimiao Liao, Xiangyang Chen, Ting Xu, Qile Jin, Zongxiu Xu, Demin Xu, Xufei Zhou, Chengming Zhu, Shouhong Guang, Xuezhu Feng

**Affiliations:** Ministry of Education Key Laboratory for Membraneless Organelles & Cellular Dynamics, Hefei National Laboratory for Physical Sciences at the Microscale, School of Life Sciences, Department of Obstetrics and Gynecology, The First Affiliated Hospital of USTC, Division of Life Sciences and Medicine, University of Science and Technology of China, Hefei, Anhui 230027, P.R. China; Ministry of Education Key Laboratory for Membraneless Organelles & Cellular Dynamics, Hefei National Laboratory for Physical Sciences at the Microscale, School of Life Sciences, Department of Obstetrics and Gynecology, The First Affiliated Hospital of USTC, Division of Life Sciences and Medicine, University of Science and Technology of China, Hefei, Anhui 230027, P.R. China; Ministry of Education Key Laboratory for Membraneless Organelles & Cellular Dynamics, Hefei National Laboratory for Physical Sciences at the Microscale, School of Life Sciences, Department of Obstetrics and Gynecology, The First Affiliated Hospital of USTC, Division of Life Sciences and Medicine, University of Science and Technology of China, Hefei, Anhui 230027, P.R. China; Ministry of Education Key Laboratory for Membraneless Organelles & Cellular Dynamics, Hefei National Laboratory for Physical Sciences at the Microscale, School of Life Sciences, Department of Obstetrics and Gynecology, The First Affiliated Hospital of USTC, Division of Life Sciences and Medicine, University of Science and Technology of China, Hefei, Anhui 230027, P.R. China; Ministry of Education Key Laboratory for Membraneless Organelles & Cellular Dynamics, Hefei National Laboratory for Physical Sciences at the Microscale, School of Life Sciences, Department of Obstetrics and Gynecology, The First Affiliated Hospital of USTC, Division of Life Sciences and Medicine, University of Science and Technology of China, Hefei, Anhui 230027, P.R. China; Ministry of Education Key Laboratory for Membraneless Organelles & Cellular Dynamics, Hefei National Laboratory for Physical Sciences at the Microscale, School of Life Sciences, Department of Obstetrics and Gynecology, The First Affiliated Hospital of USTC, Division of Life Sciences and Medicine, University of Science and Technology of China, Hefei, Anhui 230027, P.R. China; Ministry of Education Key Laboratory for Membraneless Organelles & Cellular Dynamics, Hefei National Laboratory for Physical Sciences at the Microscale, School of Life Sciences, Department of Obstetrics and Gynecology, The First Affiliated Hospital of USTC, Division of Life Sciences and Medicine, University of Science and Technology of China, Hefei, Anhui 230027, P.R. China; Ministry of Education Key Laboratory for Membraneless Organelles & Cellular Dynamics, Hefei National Laboratory for Physical Sciences at the Microscale, School of Life Sciences, Department of Obstetrics and Gynecology, The First Affiliated Hospital of USTC, Division of Life Sciences and Medicine, University of Science and Technology of China, Hefei, Anhui 230027, P.R. China; Ministry of Education Key Laboratory for Membraneless Organelles & Cellular Dynamics, Hefei National Laboratory for Physical Sciences at the Microscale, School of Life Sciences, Department of Obstetrics and Gynecology, The First Affiliated Hospital of USTC, Division of Life Sciences and Medicine, University of Science and Technology of China, Hefei, Anhui 230027, P.R. China; CAS Center for Excellence in Molecular Cell Science, Chinese Academy of Sciences, Hefei, Anhui 230027, P.R. China; Ministry of Education Key Laboratory for Membraneless Organelles & Cellular Dynamics, Hefei National Laboratory for Physical Sciences at the Microscale, School of Life Sciences, Department of Obstetrics and Gynecology, The First Affiliated Hospital of USTC, Division of Life Sciences and Medicine, University of Science and Technology of China, Hefei, Anhui 230027, P.R. China

## Abstract

Eukaryotic cells express a wide variety of endogenous small regulatory RNAs that function in the nucleus. We previously found that erroneous rRNAs induce the generation of antisense ribosomal siRNAs (risiRNAs) which silence the expression of rRNAs via the nuclear RNAi defective (Nrde) pathway. To further understand the biological roles and mechanisms of this class of small regulatory RNAs, we conducted forward genetic screening to identify factors involved in risiRNA generation in *Caenorhabditis elegans*. We found that risiRNAs accumulated in the RNA exosome mutants. risiRNAs directed the association of NRDE proteins with pre-rRNAs and the silencing of pre-rRNAs. In the presence of risiRNAs, NRDE-2 accumulated in the nucleolus and colocalized with RNA polymerase I. risiRNAs inhibited the transcription elongation of RNA polymerase I by decreasing RNAP I occupancy downstream of the RNAi-targeted site. Meanwhile, exosomes mislocalized from the nucleolus to nucleoplasm in suppressor of siRNA *(susi)* mutants, in which erroneous rRNAs accumulated. These results established a novel model of rRNA surveillance by combining ribonuclease-mediated RNA degradation with small RNA-directed nucleolar RNAi system.

## INTRODUCTION

In eukaryotic cells, ribosomal RNAs (rRNAs) are transcribed by RNA polymerase I into a single 47S polycistronic precursor in the nucleolus, which are then processed and matured into 18S, 5.8S and 28S rRNAs; 5S rRNA is independently transcribed by RNA polymerase III in the nucleus. The processing of ribosomal RNAs is extraordinarily complicated, in which defects of any steps could induce the accumulation of erroneous rRNAs (1,2). Immature rRNA intermediates or erroneous rRNAs are degraded by multiple surveillance machineries. In the nucleus, the RNA exosome has a central role in monitoring nearly every type of transcripts produced by RNA polymerase I, II and III (RNAP I, II and III) (3). The eukaryotic nuclear RNA exosome is a 3′ to 5′ exoribonuclease complex, consisting of a 9-protein catalytically inactive core complex (EXO-9) and two catalytic subunits, RRP6 (also known as EXOSC10) and Dis3 (also known as Rrp44 or EXOSC11) (4). Erroneous rRNAs are degraded from 3′ to 5′ by the RNA exosome complex. In the cytoplasm, erroneous rRNAs can be polyuridylated and degraded from 3′ to 5′ by the cytoplasmic exoribonuclease DISL-2 (also known as SUSI-1 in *Caenorhabditis elegans*) (5,6).

rRNA-derived small RNAs have been identified in a number of organisms. In *Schizosaccharomyces pombe*, defects in TRAMP-mediated RNA surveillance system elicit the biogenesis of rRNA-siRNAs (rr-siRNAs) and reduce the levels of centromeric siRNAs (7). In *Arabidopsis*, 24- or 21-nt rDNA-derived siRNAs have been identified and the latter siRNAs are accumulated upon viral infection or the depletion of the 5′ to 3′ RNA degradation machineries (8–12). In *Neurospora crassa*, 20- to 21-nt qiRNAs are produced from aberrant rRNAs in an RNA-dependent RNA polymerase (RdRP)-dependent manner, and function in DNA damage repair (13). In *C. elegans*, 22G antisense ribosomal siRNAs (risiRNAs) are generated upon environmental stresses or improper pre-rRNA processing (5,14,15).

Small regulatory RNAs direct sequence-specific regulation of gene expression via the mechanism termed RNA interference (RNAi). Small RNAs guide the Argonaute-containing protein complex to complementary nucleic acids and modulate gene expression by a number of mechanisms, including but not limiting to RNA degradation, translation inhibition, inducing heterochromatin formation, and inhibiting transcription elongation (16,17). In *C. elegans*, siRNAs silence nuclear-localized RNAs co-transcriptionally via the Nrde pathway. The NRDE complex transports 22G siRNAs from the cytoplasm to the nucleus, inhibits RNA polymerase II during the elongation phase of transcription and induces histone H3 lysine 9 (H3K9) and histone H3 lysine 27 (H3K27) trimethylation (18–20). Similarly, the nuclear Argonaute protein NRDE-3 binds risiRNAs and translocates from the cytoplasm to the nucleolus, in which the risiRNA/NRDE complex associates with pre-rRNAs and reduces the level of pre-rRNAs (5,14,21). However, the detailed mechanism of risiRNA-mediated pre-rRNA silencing is poorly understood.

To further understand the biological roles and mechanisms of risiRNAs, in this study, we isolated a series of exosome mutants in which risiRNAs were accumulated by forward and reverse genetic screens and CRISPR–Cas9–mediated gene knockout technology. We found that the nucleolar localization of exosome was important for risiRNA suppression. Meanwhile, we developed a RNAP I transcription activity assay and demonstrated that risiRNAs guided the NRDE complex to nucleoli, the association of NRDE proteins with pre-rRNAs and the inhibition of RNAP I transcription. Interestingly, we failed to detect significant change of H3K9 and H3K27 trimethylation at rDNA locus in the presence of risiRNAs. Therefore, we concluded that cells combine ribonuclease-mediated RNA degradation with small RNA-directed nucleolar RNAi system to maintain rRNA homeostasis in *C. elegans*.

## MATERIALS AND METHODS

### Strains

Bristol strain N2 was used as the standard wild-type strain. All strains were grown at 20°C unless specified. The strains used in this study are listed in Supplementary Table S2.

### Genetic screening

Genetic screening experiment was conducted as previously described (5). Briefly, to identify the factors which negatively regulate endo-siRNA generation, we searched for mutants that redistributed NRDE-3 from the cytoplasm to the nucleus in *eri-1(mg366);gfp::nrde-3* animals. NRDE-3 transports siRNAs from the cytoplasm to the nucleus. NRDE-3 localizes to the nucleus when it binds to siRNAs but accumulates in the cytoplasm in the absence of siRNA ligands, for example, in the *eri-1* mutant (20). The production of risiRNAs in *susi* mutants triggers the accumulation of NRDE-3 in the nucleus and nucleoli. *eri-1(mg366);gfp::nrde-3* animals were mutagenized by ethyl methanesulfonate (EMS), followed by a clonal screening. The F2 progeny worms were visualized under fluorescence microscope at the L3/L4 stage. Mutants that redistributed NRDE-3 to the nuclei of seam cells were selected. *susi-5* was identified by snp-SNP mapping followed by the re-sequencing of the mutant genome.

### Construction of plasmids and transgenic strains

For in situ transgene *3xflag::gfp::rpoa-2*, the 3xFLAG::GFP coding region was PCR amplified from YY174 genomic DNA with the primers 5′-ATGGACTACAAAGACCATGACGG-3′ and 5′- AGCTCCACCTCCACCTCCTTTGTATAGTTCATCCATGCCATGT-3′. A 1.5kb homologous left arm was PCR amplified with the primers 5′-GGGTAACGCCAGCACGTGTGGTCAATGTCTAACAGCCAGCGAC-3′ and 5′- TCATGGTCTTTGTAGTCCATTATGTCGCAGTCCATCGCCTGA-3′. A 1.5kb homologous right arm was PCR amplified with the primers 5′-AAGGAGGTGGAGGTGGAGCTATGGACTGCGACATAGCGTCG-3′ and 5′- GAGTGAGCTGATACCAGCGGATGTACTTTGGCAACTTTAACAAATTG-3′. And the backbone was PCR amplified from the plasmid pCFJ151 with the primers 5′-CACACGTGCTGGCGTTACCC-3′ and 5′-CCGCTGGTATCAGCTCACTCAA-3′. All these fragments were joined together by Gibson assembly to form the *3xflag::gfp::rpoa-2* plasmid with the ClonExpress MultiS One Step Cloning Kit (Vazyme Biotech, Nanjing, China, Cat. No. C113-01/02). This plasmid was co-injected into N2 animals with three sgRNA expression vectors, rpoa-2_sgRNA#1, rpoa-2_sgRNA#2, rpoa-2_sgRNA#3, 5 ng/μl pCFJ90 and Cas9 II expressing plasmid. Primer pairs for constructing sgRNA expression vectors are shown in Supplementary Table S3.

For in-situ transgene *mCherry::rpoa-2*, the mCherry fragment was amplified with the primers 5′-ATGGTCTCAAAGGGTGAAGAAG-3′ and 5′-ATAGCTCCACCTCCACCTCCCTTATACAATTCATCCATGCCACC-3′ and the vector plasmid was amplified with the primers 5′-GGAGGTGGAGGTGGAGCTATGGACTGCGACATAGCGTC-3′ from the *gfp::rpoa-2* plasmid. The two fragments were joined together by Gibson assembly to form the *mCherry::rpoa-2* repair plasmid. CRISPR plasmid mixture containing 30ng/μl rpoa-2_sgRNA#1, 30ng/μl rpoa-2_sgRNA#2, 30ng/μl rpoa-2_sgRNA#3, 50 ng/μl Cas9 II expressing plasmid and 5 ng/μl pCFJ90 was co-injected into N2 animals.

For in-situ transgene *3xflag::gfp::nrde-2*, the *3xflag* fragment was amplified with the primers 5′-ATGGACTACAAAGACCATGAC-3′ and 5′- ATAGCTCCACCTCCACCTCCTTTGTATAGTTCATCCATGCC-3′ from YY174 genomic DNA. A 1.5kb homologous left arm was PCR amplified with the primers 5′-GGGTAACGCCAGCACGTGTGGTCAATGTCTAACAGCCAGCGAC-3′ and 5′-TCATGGTCTTTGTAGTCCATATACGCTCGAAACATTGTTCATTA-3′. A 1.5kb homologous right arm was PCR amplified with the primers 5′-GGAGGTGGAGGTGGAGCTATGTTTCGAGCGTATGGAAATAATG-3′ and 5′-GCGGATAACAATTTCACCTAGATTATCCGAATCGTTTGCTAGAAC-3′. The backbone was PCR amplified with the primers 5′-TAGGTGAAATTGTTATCCGCTGG-3′ and 5′-TATTTCACACCGCATATGGTGC-3′from pCFJ151. All these fragments were joined together by Gibson assembly to form the 3xflag::gfp::nrde-2 plasmid. This plasmid was co-injected into N2 animals with two sgRNA expression vectors, nrde-2_sgRNA#1, nrde-2_sgRNA#2 and Cas9 II expressing plasmid. Primer pairs for constructing sgRNA expression vectors are shown in Supplementary Table S3.

For the constructing of *mcherry::dis-3*, a 2 kb promoter region was amplified with the primers 5′- CGACTCACTAGTGGGCAGATATCGTCGTGATTATCCATTTTTGAAAC-3′ and 5′- TCTTCACCCTTTGAGACCATGACGTTCAAATCCATACCTTC′. The *dis-3* CDs region and 3′ UTR region were amplified as a whole fragment with the primers 5′-GGAGGTGGAGGTGGAGCTATGGATTTGAACGTCAAACAAAG-3′ and 5′- GGCCTTGACTAGAGGGTACCAGCCGTCCCTATTGGATGATAAAT-3′. The *mCherry* coding sequence was amplified from PFCJ90 with 5′-AGCTCCACCTCCACCTCCCTTATACAATTCATCCATGCC-3′ and 5′-ATGGATTTGAACGTCATGGTCTCAAAGGGTGAAGAAGA-3′. The linearized backbone was amplified from PCFJ151 with primers 5′-ATCTGCCCACTAGTGAGTCG-3′ and 5′-GGTACCCTCTAGTCAAGGCC-3′. The transgene was integrated onto the *C. elegans*' chromosome III of the strain EG8080 by MosSCI technology (22).

For *3xflag::gfp::exos-1*, a 2 kb promoter region was amplified with the primers 5′- CGACTCACTAGTGGGCAGATTGCCTGACCTTAAGGCGG-3′ and 5′-TCATGGTCTTTGTAGTCCATCGTTTCGGCGAGCATTTTCT-3′. The *exos-1* CDs region and 3′ UTR region was amplified as a whole fragment with the primers 5′-AAGGAGGTGGAGGTGGAGCTATGCTCGCCGAAACGCTTGT-3′ and 5′- GGCCTTGACTAGAGGGTACCCAGTGAGCCCATCTCATCAT-3′. The *3xflag::gfp* coding sequence was amplified from YY174 genomic DNA with 5′-ATGCTCGCCGAAACGATGGACTACAAAGACCATGACGGTG-3′ and 5′-AGCTCCACCTCCACCTCCTTTGTATAGTTCATCCATGC-3′. The linearized backbone was amplified from pCFJ151 with primers 5′-ATCTGCCCACTAGTGAGTCG-3′ and 5′-GGTACCCTCTAGTCAAGGCC-3′. The transgene was integrated onto the *C. elegans*' chromosome II of the strain EG4322 by MosSCI technology.

For *3xflag::gfp::exos-2*, a 2kb promoter region was amplified with the primers 5′- CGACTCACTAGTGGGCAGATACGAGAACAATCAAAGCAACG-3′ and 5′- TCATGGTCTTTGTAGTCCATGGTGACTTCGAAACTCATTT-3′. The *exos-2* CDS region and 3′ UTR region were amplified as a whole fragment with the primers 5′- AAGGAGGTGGAGGTGGAGCTATGAGTTTCGAAGTCACCGG-3′ and 5′- GGCCTTGACTAGAGGGTACCCGGTACCAACAACTCCAACG-3′. The *3xflag::gfp* coding sequence was amplified from YY174 genomic DNA with 5′-ATGGACTACAAAGACCATGACG-3′ and 5′-AGCTCCACCTCCACCTCCTTTGTATAGTTCATCCATGCCA-3′. The linearized backbone was amplified from pCFJ151 with primers 5′-ATCTGCCCACTAGTGAGTCG-3′ and 5′-GGTACCCTCTAGTCAAGGCC-3′. The transgene was integrated onto the *C. elegans*' chromosome II of the strain EG4322 by MosSCI technology.

*exos-10* locates in the operon CEOP2496. For the constructing of *3xflag::gfp::exos-10*, a 2.1 kb promoter region was PCR amplified with the primers 5′-CGACTCACTAGTGGGCAGATCAACGTCGGACTTCTCGAAT-3′ and 5′-CATATCTTGATAATCGTCCTCAT-3′ from N2 genomic DNA. A transpliced sequence was amplified with the primers 5′- AGGACGATTATCAAGATATGATGACGACATGCACTTTATA-3′ and 5′-TTCTTCTCCTGACATTCTGTAAAT-3′. The 3xFLAG::GFP coding region was PCR amplified from YY174 genomic DNA with the primers 5′-ACAGAATGTCAGGAGAAGAAGACTACAAAGACCATGACGGT-3′ and 5′-ATTGATTCTTCTCCTGACATAGCTCCACCTCCACCTCCT-3′. The EXOS-10 coding region and 3′ UTR region were PCR amplified with the primers 5′-ATGTCAGGAGAAGAATCAATGC-3′ and 5′-GGCCTTGACTAGAGGGTACCTGGATCTGAAGCTTAACCTATTC-3′. The pCFJ151 vector fragment was PCR amplified with the primers 5′-GGTACCCTCTAGTCAAGGCC-3′ and 5′-ATCTGCCCACTAGTGAGTCG-3′ from the pCFJ151 plasmid. These five fragments were joined together by Gibson assembly to form the *gfp::exos-10* repair plasmid. The transgene was integrated onto the *C. elegans*' chromosome II by MosSCI technology.

For *rrp-8::mCherry*, the promoter region was PCR amplified with the primers 5′- CCTGTCAATTCCCAAAATACTTGGAAAGCATTTTCAGGCG-3′ and 5′- GAAAAATTCAACGGAATGCTCTGAAATTGTTAACACAGATGATAAAAG-3′ and the coding region was PCR amplified with the primers 5′-AGCATTCCGTTGAATTTTTCGCTG-3′ and 5′-CAGCTCCACCTCCACCTCCGCGTTTCTTATACAAACAAGGC-3′ from N2 genomic DNA respectively. The mCherry fragment was PCR amplified with the primers 5′-CGGAGGTGGAGGTGGAGCTGTCTCAAAGGGTGAAGAAGATAAC-3′ and 5′- ACAAAAAATCAAAAAATCACTTATACAATTCATCCATGCCACC-3′ from the plasmid pCFJ90. The primers 5′-TGATTTTTTGATTTTTTGTTGATTT-3′ and 5′-TTCAAAGAAATCGCCGACTTCAATCGCTCTCAACGTTTCTG-3′ were used to generate the 3′ UTR region of *rrp-8*. The vector fragment was PCR amplified with the primers 5′- AGAAACGTTGAGAGCGATTGGTGAGTTCCAATTGATAATTGTGAT-3′ and 5′-GTATTTTGGGAATTGACAGGG-3′ from plasmid pSG274. These five fragments were joined together by Gibson assembly to form the *rrp-8::mCherry* repair plasmid. The transgene was integrated onto the *C. elegans*' chromosome I via a modified counterselection (cs)-CRISPR method (23).

For *rbd-1::mCherry*, the DNA region containing the promoter and CDS sequence was PCR amplified with the primers 5′-CTCGAGGAATTCCTGCAGGAGGCTGATTGACCAGCGCAACA-3′ and 5′- ATAGCTCCACCTCCACCTCCATCCTTTTCATCATCGGCAATTTG-3′ from N2 genomic DNA. The mCherry fragment was PCR amplified with the primers 5′-GGAGGTGGAGGTGGAGCTATGGTCTCAAAGGGTGAAGAAGA-3′ and 5′- CTTATACAATTCATCCATGCCA-3′ from the plasmid pCFJ90. The primers 5′-GCATGGATGAATTGTATAAGTAATTGTTATTTTTGCCTGTTTCTGTTA-3′ and 5′- AAGCTTCGTGGATCCAGATAATCCGCCGCAATGCCATTTTTCTG-3′ were used to generate the 3′ UTR region of *rbd-1*. The vector fragment was PCR amplified with the primers 5′-TATCTGGATCCACGAAGCTT-3′ and 5′-TCCTGCAGGAATTCCTCGAG-3′ from plasmid pSG274. These four fragments were joined together by Gibson assembly to form the *rbd-1::mCherry* repair plasmid. The transgene was integrated onto the *C. elegans*' chromosome I via a modified counterselection (cs)-CRISPR method (23).

The sgRNAs used in this study for transgene construction are listed in Supplementary Table S3.

### CRISPR/Cas9-mediated gene deletion

Multiple sgRNAs-guided chromosome deletion was conducted as previously described (24). To construct sgRNA expression plasmids, the 20 bp *unc-119* sgRNA guide sequence in the pU6::*unc-119* sgRNA(F + E) vector was replaced with different sgRNA guide sequences as described previously. Addgene plasmid #47549 was used to express Cas9 II protein. Plasmid mixtures containing 30 ng/μl of each of the three sgRNA expression vectors, 50 ng/μl Cas9 II expressing plasmid, and 5 ng/μl pCFJ90 were co-injected into YY178: *eri-1(mg366);3xflag::gfp::nrde-3(ggIS1)* animals. The deletion mutants were screened by PCR amplification and confirmed by sequencing. The sgRNAs used in this study are listed in Supplementary Table S3.

### RNAi

RNAi experiments were conducted as previously described (25). All the RNAi experiments were started by placing the synchronized embryos on the seeded RNAi plates. Worms grew up to expected stage on RNAi plates and were collected. HT115 bacteria expressing the empty vector L4440 (a gift from A. Fire) was used as controls. Bacterial clones expressing dsRNA were obtained from the Ahringer RNAi library and were sequenced to verify their identity. The 18S RNAi #1 clone with dsRNA targeting the 18S rRNA was PCR amplified with the primer pairs 5′- CGCAATTTGCGTCAACTGTGG-3′ and 5′-TCTTCTCGAATCAGTTCAGTCC-3′ from N2 genomic DNA. The L4440 vector fragment was amplified with the primer 5′-ACTGAACTGATTCGAGAAGActtgatatcgaattcctgcagc-3′ and 5′-CACAGTTGACGCAAATTGCGCTTATCGATACCGTCGACCTC-3′. These two fragments were joined together to generate the dsRNA expression plasmid targeting 18S rRNA. The 18S RNAi #2 clone with dsRNA targeting the 18S rRNA was PCR amplified with the primer pairs 5′-TCTATCCGGAAAGGGTGTCTGC-3′ and 5′- CACTCCACCAACTAAGAACGGC-3′ from N2 genomic DNA. The L4440 vector fragment was amplified with the primer 5′- CGTTCTTAGTTGGTGGAGTGcttgatatcgaattcctgcagcc-3′ and 5′-AGACACCCTTTCCGGATAGActtatcgataccgtcgacctcga-3′. These two fragments were joined together to generate the dsRNA expression plasmid targeting 18S rRNA.

### Chromatin immunoprecipitation (ChIP)

All the RNAi experiments were started by placing the bleached embryos on the seeded RNAi plates. Worms grew up to gravid adult stage on RNAi plates were collected and bleached to harvest embryos for the RNA immunoprecipitation (RIP) assay and the histone ChIP assay. For the RPOA-2 ChIP assay, young adults were collected. 25 μl worms were used in each replicate and at least 3 biological replicates were performed for each experiment.

ChIP experiments of H3K9me3 or H3K27me3 were performed as previously described with hypochlorite-isolated embryos (19). Briefly, after crosslinking, samples were sonicated 23 cycles (each cycle: 30 s on and 30 s off) with a Bioruptor UCD-200 (Diagenode). Lysates were precleared with agarose beads (BBI no. C600957-0005) and then immunoprecipitated with 2 μl anti-trimethylated H3K9 antibody (Millipore no. 07-523) or 2 μl anti-trimethylated H3K27 antibody (Millipore no. 07-449). ChIP signals were normalized to levels of *eft-3* and the data were expressed as ratios of indicated animals exposed to ± dsRNA.

For Pol I transcription, ChIP experiments were performed with young adults. After cross-linking, samples were resuspended in FA buffer (50 mM Tris/HCl at pH 7.5, 1 mM EDTA, 1% Triton X-100, 0.1% sodium deoxycholate, 150 mM NaCl) containing proteinase inhibitor tablet (Roche, 04693116001) and sonicated for 23 cycles at medium output (each cycle: 30 seconds on and 30 seconds off) with a Bioruptor 200. Lysates were precleared and then immunoprecipitated with 1.5 μl of anti-GFP antibody (Abcam, ab290) for GFP::RPOA-2 overnight at 4°C. Antibody-bound complexes were recovered with Dynabeads Protein A. DNA was treated with RNase (Roche) and Proteinase K (New England Biolabs).

Quantitative real-time PCR (qPCR) was performed using an MyIQ2 machine (Bio-Rad) with SYBR Green Master Mix (Vazyme, Q111-02). The primers used in this work are listed in Supplementary Table S4.

### Deep sequencing of small RNAs and bioinformatic analysis

Deep sequencing of small RNAs and bioinformatic analysis were conducted as previously described (14). Briefly, total RNAs were isolated from L3 stage worm using a dounce homogenizer (pestle B) in TRIzol solution (Invitrogen) followed by DNase I digestion (Fermentas, no. 18068015). 3xFLAG::GFP::NRDE-3-associated siRNAs were isolated from L3 stage worm lysates as described previously (5,20). The lysate was pre-cleared with protein G-agarose beads (Roche) and incubated with anti-FLAG M2 agarose beads (Sigma #A2220). The beads were washed extensively and were eluted with 100 μg/ml 3xFLAG peptide (Sigma #F4799). The eluates were incubated with TRIzol reagent followed by isopropanol precipitation and DNase I digestion (Fermentas). To facilitate 5′-phosphate-independent deep sequencing, the precipitated RNAs were treated with calf intestinal alkaline phosphatase (CIAP, Invitrogen), re-extracted with TRIzol, and treated with T4 polynucleotide kinase (T4 PNK, New England Biolabs) in the presence of 1 mM ATP.

Small RNAs were subjected to deep sequencing using an Illumina platform (Novogene Bioinformatics Technology Co., Ltd). Briefly, small RNAs ranging from 18 to 30 nt were gel-purified and ligated to a 3′ adaptor (5′-pUCGUAUGCCGUCUUCUGCUUGidT-3′; p, phosphate; idT, inverted deoxythymidine) and a 5′ adaptor (5′-GUUCAGAGUUCUACAGUCCGACGAUC-3′). The ligation products were gel-purified, reverse transcribed, and amplified using Illumina's sRNA primer set (5′-CAAGCAGAAGACGGCATACGA-3′; 5′-AATGATACGGCGACCACCGA-3′). The samples were then sequenced using an Illumina Hiseq platform.

The Illumina-generated raw reads were first filtered to remove adaptors, low quality tags and contaminants to obtain clean reads at Novogene. Clean reads ranging from 18 to 30 nt were mapped to the unmasked *C. elegans* genome and the transcriptome assembly WS243, respectively, using Bowtie2 (26) with default parameters. The number of reads targeting each transcript was counted using custom Perl scripts and displayed by IGV (27). The number of total reads mapped to the genome minus the number of total reads corresponding to sense rRNA transcripts (5S, 5.8S, 18S and 26S) and sense protein coding mRNA reads was used as the normalization number to exclude the possible degradation fragments of sense rRNAs and mRNAs.

### RNA immunoprecipitation (RIP) assay and rRNA quantification

3xFLAG::GFP::NRDE-2- and 3xFLAG::GFP::NRDE-3-associated pre-rRNAs were isolated from embryo lysates as described previously (5,20). 20 μl embryos were used in each replicate and 3 biological replicates were performed for each experiment. The lysate was pre-cleared with protein G-agarose beads (Roche) and incubated with anti-FLAG M2 Magnetic Beads (Sigma #M8823). The beads were washed extensively and were eluted with 100 μg/ml 3xFLAG peptide (Sigma #F4799). The eluates were incubated with TRIzol reagent followed by isopropanol precipitation and DNase I digestion (Fermentas).

RNA was reverse transcribed via GoScript™ Reverse Transcription System (Promega #A5001). Quantitative real-time PCR (qPCR) was performed using an MyIQ2 machine (Bio-Rad) with SYBR Green Master Mix (Vazyme, Q111-02). The primers used in this work are listed in Supplementary Table S5.

### Actinomycin D treatment

Actinomycin D (MedChemExpress no. HY-17559, CAS:50-76–0) was prepared to 20 mg/ml in DMSO as stock solution. The actinomycin D stock solution was diluted to 5 or 10 μg/ml with concentrated OP50. NGM plates were prepared and placed at room temperature overnight before use. Embryos were placed onto the seeded plates and grew to young adults before collection for ChIP.

### Quantitative real-time PCR

All quantitative real-time PCR (qPCR) experiments were performed using an MyIQ2 machine (Bio-Rad). DNA or cDNA was quantified with SYBR Green Master Mix (Vazyme, Q111-02) and the qPCR reactions were performed according to the vendor's instructions. RNA was first digested by DNase I (Fermentas), followed by isopropanol precipitation, and then reverse transcribed via GoScript™ Reverse Transcription System (Promega #A5001) with random primers. The primer sequences for ChIP-qPCR and cDNA detection are listed in Supplementary Tables S4 and S5. The numbers of replicates are indicated in the figure legends.

### Imaging

Images were collected using Leica DM4 microscopes. The nuclear translocation of GFP::NRDE-3 is scored as positive if at least one of the seam cells in an animal has a nuclear localized GFP::NRDE-3. *C. elegans* has sixteen seam cells at L4 larva. GFP::NRDE-3 accumulates in the nuclei in most of the seams cells in *susi* mutants. A positive cell usually displays a significantly brighter GFP::NRDE-3 in the nucleus than in the cytoplasm, which can be easily distinguished by fluorescent visualization. However, we did observe that GFP::NRDE-3 accumulates in the cytoplasm in a number of seam cells in the mutant animals, because of some unknown stochastic reasons.

Images show seam cells of the indicated L2 or L4 stage animals expressing GFP::NRDE-3.

### Brood size

Progenies that reach L4 or young adult stages were counted.

### Statistics

Bar graphs with error bars are presented with mean and standard deviation. All of the experiments were conducted with independent *C. elegans* animals for the indicated N times. Statistical analysis was performed with two-tailed Student's *t*-test.

## RESULTS

### Genetic screening identified risiRNAs that accumulated in the *susi-5(ceDis3)* mutant

**Figure 1.**
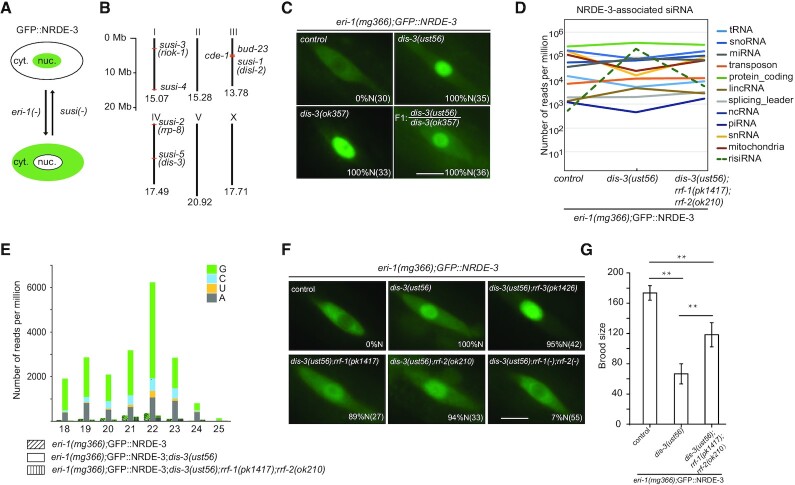
A genetic screening identified the accumulation of antisense ribosomal siRNAs (risiRNAs) in *dis-3* mutants. (**A**) The subcellular localization of NRDE-3 was used as an indicator to search for suppressors of endo-siRNA generation. cyt., cytoplasm; nuc., nucleus. (**B**) Summary of *susi* genes identified by forward genetic screening in *C. elegans* (5,14). Numbers indicate the size of each chromosome. (**C**) Images show seam cells of L4 stage animals expressing GFP::NRDE-3. Numbers indicate the percentage of animals with nucleus-enriched NRDE-3 in seam cells (% N). The number of scored animals is indicated in parentheses. Scale bars, 5 μm. (**D**) Results of the deep sequencing of NRDE-3-associated siRNAs from L3 stage animals. The green dashed lines indicate risiRNAs. The sense ribosomal reads and the sense protein-coding reads are discarded. (**E**) Size distribution and 5′-end nucleotide preference of NRDE-3-associated risiRNAs in L3 animals. (**F**) Images show seam cells of the respective L4 stage animals expressing GFP::NRDE-3, labeled as in (**C**). Scale bars, 5 μm. (**G**) Brood size of indicated animals grown at 20°C. Data are presented as the mean ± s.d.; *n* ≥ 15 animals; **P < 0.01.

The nuclear Argonaute protein NRDE-3 transports siRNAs from the cytoplasm to the nucleus and remains in the nucleus when it binds to siRNAs but resides in the cytoplasm in the absence of siRNA ligands, as observed in the wild-type and *eri-1(–)* animals respectively in *C. elegans* (20). The subcellular localization confers NRDE-3 a convenient tool to monitor the abundance of cellular siRNAs (20). We previously described a forward genetic screening to search for suppressor of siRNA (*susi)* genes based on altered subcellular localization of NRDE-3 in *C. elegans* (Figure 1A) (5,14). This screening identified a cytoplasmic localized exoribonuclease, SUSI-1(ceDis3L2), and a number of rRNA modifying and processing enzymes, including SUSI-2(ceRRP8) (Figure 1B). *susi-1* is the homologue of human DIS3-like exonuclease 2 (Dis3L2), therefore, the name *disl-2* is used hereafter*. susi-2* is the homologue of yeast RRP8, the name *rrp-8* is used hereafter. Here, we reported that this screening identified a mutant allele, *ust56*, in *susi-5(ceDis3)* gene (see below) that suppressed risiRNA production. In *eri-1(mg366);susi-5(ust56)* mutants, the Argonaute protein NRDE-3 accumulated in the nucleus of seam cells in *C. elegans* (Figure 1C).

To determine the molecular identity of *susi-5, w*e mapped *susi-5(ust56)* to the open reading frame C04G2.6 by SNP mapping followed by genome resequencing. C04G2.6 is predicted to encode a protein that is homologous to yeast DIS3 and human RRP44 and engages in pre-rRNA surveillance (28). C04G2.6 has a PIN domain, two cold shock domains (CSD), an RNB domain and S1 domain (Supplementary Figure S1A). While the CSDs and the S1 domains contribute to RNA binding, both the RNB and PIN domains are responsible for target RNA degradation (29). In the *ust56* allele, a conserved amino acid in the cold shock domain, Arg363, was mutated to cysteine (Supplementary Figure S1A). We acquired one additional allele, *dis-3(ok357)*, from the Caenorhabditis Genetics Center (CGC). NRDE-3 also accumulated in the nucleus of seam cells in the *eri-1(mg366);dis-3(ok357)* strain (Figure 1C). An ectopically expressed mCherry::DIS-3 transgene rescued the cytoplasmic localization of NRDE-3 in *eri-1(mg366);susi-5(ust56)* animals (Supplementary Figure S1B). Thus, we concluded that *susi-5* is *dis-3*, and the name *dis-3* is used hereafter. The *ok357* mutation deleted the PIN and two CSD domains and is a null allele (Supplementary Figure S1A). The *dis-3(ok357)* mutant is arrested at the larva stage and has no progeny (Supplementary Figure S1C). However, the *dis-3(ust56)* strain is fertile, and approximately 180 progenies are produced per hermaphrodite at 20°C, suggesting that the R363C mutation partially disrupts DIS-3 function. In addition, *dis-3(ust56)* is a temperature sensitive allele. At 25°C, the *dis-3(ust56)* mutant is sterile. In this study, we used *dis-3(ust56)* as the reference allele to study the biogenesis and function of risiRNAs.

The mutation of *susi* genes results in the production of risiRNAs and the accumulation of NRDE-3 in nuclei and nucleoli in an *eri-1*-independent manner (5,14). We deep sequenced total small RNAs in control animals and the *dis-3(ust56)* mutants and observed an increase in risiRNAs as well (Supplementary Figure S2A). To confirm that NRDE-3 associated with risiRNAs in *dis-3* mutants, we also immunoprecipitated NRDE-3 from *dis-3(ust56)* animals and deep sequenced NRDE-3-associated small RNAs. risiRNAs were enriched in *dis-3(ust56)* mutants compared to the level found in control strains (Figure 1D and Supplementary Figure S2B). risiRNAs belong to the 22G-RNA category in *C. elegans*. The majority of risiRNAs start with a guanosine at the 5′-end and are 22 nt in length (Figure 1E). The generation of risiRNAs required two RNA-dependent RNA polymerases, RRF-1 and RRF-2, which are essential for the production of 22G-RNAs (Supplementary Figure S2C). In *rrf-1;rrf-2* double mutants, NRDE-3 bound substantially fewer risiRNAs (Figure 1E and Supplementary Figure S2B) and accumulated in the cytoplasm (Figure 1F). The presence of risiRNAs decreased the fertility of *C. elegans*. While the *dis-3(ust56)* mutation reduced the brood size of animals, the *rrf-1* and *rrf-2* mutations partially restored the strain fecundity (Figure 1G). Interestingly, *eri-1;dis-3(ust56)* double mutant has a more severe fertility defect compared to *dis-3(ust56)* alone (Figure 1G versus Supplementary Figure S1C). A possible reason is that *dis-3* is required for 18S, 5.8S and 26S rRNA processing and *eri-1* is required for 5.8S rRNA processing. In the double mutants, the quality and quantity of rRNAs are likely more severely compromised.

Therefore, we concluded that *dis-3* is a *susi* gene that suppressed risiRNA production.

### *exosome* acts in suppressing risiRNA generation

**Figure 2.**
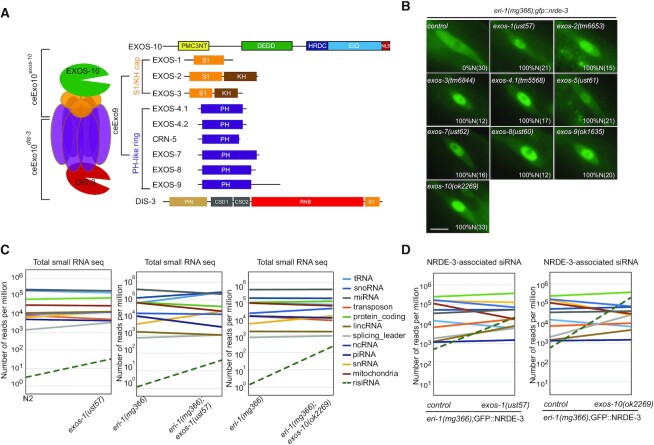
risiRNAs were enriched in exosome mutants. (**A**) Schematics of the exosome complex and the subunits in *C. elegans*. (**B**) Images show seam cells of the respective L2 stage animals expressing GFP::NRDE-3. Numbers indicate the percentage of animals with nucleus-enriched NRDE-3 in seam cells (% N). The number of scored animals is indicated in parentheses. Scale bars, 5 μm. Schematics of the alleles are shown in Supplementary Figure S3. (**C**) Results from the deep sequencing of total small RNAs from L3 animals. The green dashed lines indicate risiRNAs. (D) Results from the deep sequencing of NRDE-3-associated siRNAs from L3 animals.

DIS-3 is a core factor of the RNA exosome, which is a 3′ to 5′ exoribonuclease complex containing a 9-protein catalytically inactive core complex (EXO-9) and two catalytic active subunits, EXOS-10 and DIS-3 (Figure 2A) (28). EXO-9 forms a double-layered barrel-like structure that comprises six ribonuclease (RNase) pleckstrin homology (PH)-like proteins (EXOS-4.1, EXOS-4.2, CRN-5, EXOS-7, EXOS-8 and EXOS-9) and three S1/K homology (KH) ‘cap’ proteins (EXOS-1, EXOS-2 and EXOS-3). All these factors are conserved from yeast to humans. Most of the exosome subunits are essential, and loss-of-function mutations in them led to larval development arrest or animal sterility at 20°C (Supplementary Figure S3A) (14). To determine whether other components of the exosome complex, in addition to DIS-3, are also involved in suppressing risiRNA production, we acquired the mutants of *exos-2(tm6653*), *exos-3(tm6844), exos-4.1(tm5568), exos-9(ok1635)* and *exos-10(ok2269)* from the National Bioresource Project and the CGC, and also generated *exos-1(ust57)*, *exos-5(ust61), exos-7(ust62)* and *exos-8(ust60)* by CRISPR/Cas9-mediated gene deletion (Supplementary Figure S3B-J). In all of the mutants, NRDE-3 accumulated in the nucleus in seam cells (Figure 2B). Since *exos-1(ust57)* and *exos-10(ok2269)* homozygous mutants can still generate a few progenies, we deep sequenced total small RNAs and NRDE-3-associated small RNAs in control animals and *eri-1(mg366);exos-1(ust57)* and *eri-1(mg366);exos-10(ok2269)* mutants at L3 stage and observed an increase in the levels of both total risiRNAs and NRDE-3-associated risiRNAs (Figure 2C and D). Thus, we concluded that the exosome complex is involved in the suppression of risiRNA production.

Exosome plays essential roles in the processing and maturing of ribosomal RNAs by trimming pre-rRNA intermediates and eliminating rRNA by-products with the assistance of multiple cofactors. In yeast, when Rrp44(DIS-3) was impaired, the catalytically inactive exosome subunits and exosome cofactor Mtr4 interact with increased 5′ external transcribed spacers (5′ ETS) or 3′ ends of 5.8S precursors (30). In human, both DIS-3 and RRP6 are involved in the 3′-end maturation and 5′ ETS removal of 5.8S rRNAs (31). In *Arabidopsis*, the disruption of Exo9 or downregulating Rrp44(DIS-3) lead to the accumulation of 5′ ETS and 5.8S rRNA precursors with untrimmed 3′ ends as well (32).

**Figure 3.**
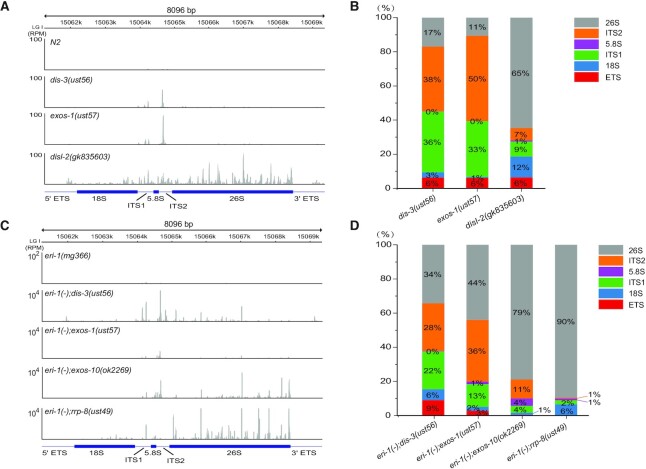
risiRNAs were derived from the untrimmed parts of erroneous rRNAs in exosome mutants. (**A**) An overview of the risiRNA distribution in total small RNA deep sequencing data from the indicated animals, as shown by IGV. (**B**) The proportion of risiRNAs derived from different regions of rDNA loci according to the total small RNA deep sequencing data. (**C**) An overview of the risiRNA distribution of NRDE-3-associated siRNA deep sequencing data from the indicated animals. (**D**) The proportion of risiRNAs derived from different regions of rDNA loci according to the NRDE-3-associated siRNA deep sequencing data.

Our previous work showed that erroneous ribosomal RNAs promoted the generation of risiRNAs (14). Additionally, when the cytoplasm-localized 3′-5′ exoribonuclease DISL-2 was mutated, both risiRNAs and 3′ ends-oligouridylated 26S rRNAs increased (5). Here, we deep sequenced both total small RNAs and NRDE-3-associatd siRNAs and compared the distribution of risiRNAs along the rDNA locus between the exosome *(dis-3, exos-1* and *exos-10)*, *disl-2* and *rrp-8* mutants. RRP-8 is responsible for the m1A modification at the A674 site of 26S rRNAs and its loss results in risiRNA accumulation (14). In *disl-2* mutant, 65% of the risiRNAs mapped to 26S rRNA region (Figure 3A-B). Yet in the *dis-3(ust56)* and *exos-1(ust57)* animals, a large fraction of risiRNAs mapped to the internal transcribed spacers (ITS1 or ITS2) region. In *eri-1;rrp-8* mutant, 90% of the NRDE-3-associated risiRNAs mapped to 26S rRNA region (Figure 3C and D). Yet in the *eri-1;dis-3* and *eri-1;exos-1* mutant, a large fraction of NRDE-3-associated risiRNAs mapped to the internal transcribed spacers (ITS1 or ITS2) region as well. This data is consistent with the characteristics of the aberrant rRNA intermediates produced during the rRNA processing and maturation steps in the designated mutants. Yet how erroneous rRNAs or intermediates are transported from the nucleoli to the cytoplasm for the RdRP-mediated risiRNA synthesis remains to be uncovered.

### risiRNAs direct the association of NRDE proteins with pre-rRNAs

**Figure 4.**
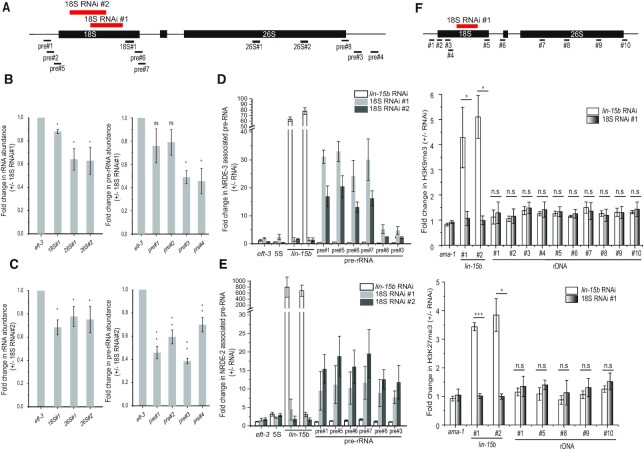
risiRNAs induced an association of NRDE proteins with pre-rRNAs. (**A**) Schematic of the rDNA transcription unit and real-time PCR primers. The two thick red bars indicate the dsRNA segments targeting 18S rRNAs. (**B**, **C**) Expression levels of rRNAs and pre-rRNAs quantified by real-time PCR after exogenous RNAi targeting 18S rRNA. (**D**, **E**) Relative amount of NRDE protein-bound pre-rRNAs by immunoprecipitating NRDE proteins followed by quantitative real time PCR of the associated RNAs. *lin-15b* is a protein coding gene, which is used as a positive control for RIP experiments. (**F**) ChIP analysis of rDNA loci upon treatment of RNAi targeting the *lin-15b* gene or 18S rRNA. Trimethylation of H3K9 and H3K27 were measured. Data are presented as mean ± s.d.; *n* = 3; *P < 0.05, **P < 0.01, ***P < 0.001, n.s., not significant.

We investigated the molecular mechanism of nucleolar RNAi. First, we fed wild-type animals with exogenous dsRNA targeting 18S rRNA and observed the downregulation of both pre-rRNAs and mature rRNAs (Figure 4A–C), which is consistent with previous report (5). Then we performed an RNA immunoprecipitation (RIP) assay to test whether risiRNAs guide NRDE complex to pre-rRNAs. We fed animals with dsRNAs targeting an mRNA *lin-15b* or dsRNAs targeting 18S rRNAs. NRDE-2 and NRDE-3 were immunoprecipitated and the associated RNAs were quantified by quantitative real time PCR. RNAi targeting *lin-15b* triggered NRDE proteins to *lin-15b* pre-mRNA and RNAi targeting 18S rRNA specifically induced the association of NRDE-2/3 with pre-rRNAs (Figure 4D and E).

Then we tested whether risiRNAs were able to induce histone modifications at rDNA loci. Small interfering RNAs have been shown to guide the NRDE complex to targeted pre-mRNAs, induce H3K9, H3K23 and H3K27 trimethylation at the corresponding genomic loci, inhibit RNAP II-mediated transcription elongation, and silence gene expression in the nucleus in *C. elegans* (18,19,33,34). To determine whether risiRNAs similarly induce histone modifications at the rDNA loci, we conducted ChIP assays with anti-H3K9me3 and anti-H3K27me3 antibodies. However, we failed to detect a significant change of the levels in H3K9 and H3K27 trimethylation at the rDNA loci in the presence of risiRNAs (Figure 4F). As a positive control, dsRNAs targeting the *lin-15b* gene, encoding an RNAP II transcript, induced both H3K9 and H3K27 trimethylation, as reported previously (19,33).

### risiRNAs inhibit RNA polymerase I-directed transcription

To determine whether risiRNA-guided nucleolar RNAi silences rRNAs by inhibiting RNAP I-directed transcription elongation, we generated GFP- and mCherry-tagged RPOA-2 transgene *in situ* by CRISPR/Cas9 technology. RPOA-2 is the core subunit of RNAP I and contributes to the polymerase activity (35). Knocking down RPOA-2 by RNAi caused sterility in the animals, suggesting that RPOA-2 played essential roles (Supplementary Figure S4A). RPOA-2 was enriched in the nucleoli and colocalized with the nucleoli marker FIB-1 (Supplementary Figure S4B and C), a finding that was consist with their functions in rRNA transcription. In 1- to 8-cell embryos, in which rDNA is not actively transcribed, FIB-1 foci was absent and RPOA-2 was evenly distributed in the nucleus without significant nucleolar enrichment (Supplementary Figures S4D and S5A). Actinomycin D is able to block the transcriptional of both RNAP I and RNAP II (36). Actinomycin D treatment did not substantially change the expression levels and subcellular location of RPOA-2 and FIB-1 (Supplementary Figure S5A–C). Similarly, actinomycin D treatment did not change the subcellular localization of NRDE-3 and EXOS-10 (Supplementary Figure S6A and B). These data suggested that actinomycin D treatment did not significantly change the nucleolar structure and risiRNA expression. Western blotting also showed that the addition of actinomycin D did not significantly change the protein level of GFP::RPOA-2 (Supplementary Figure S6C).

**Figure 5.**
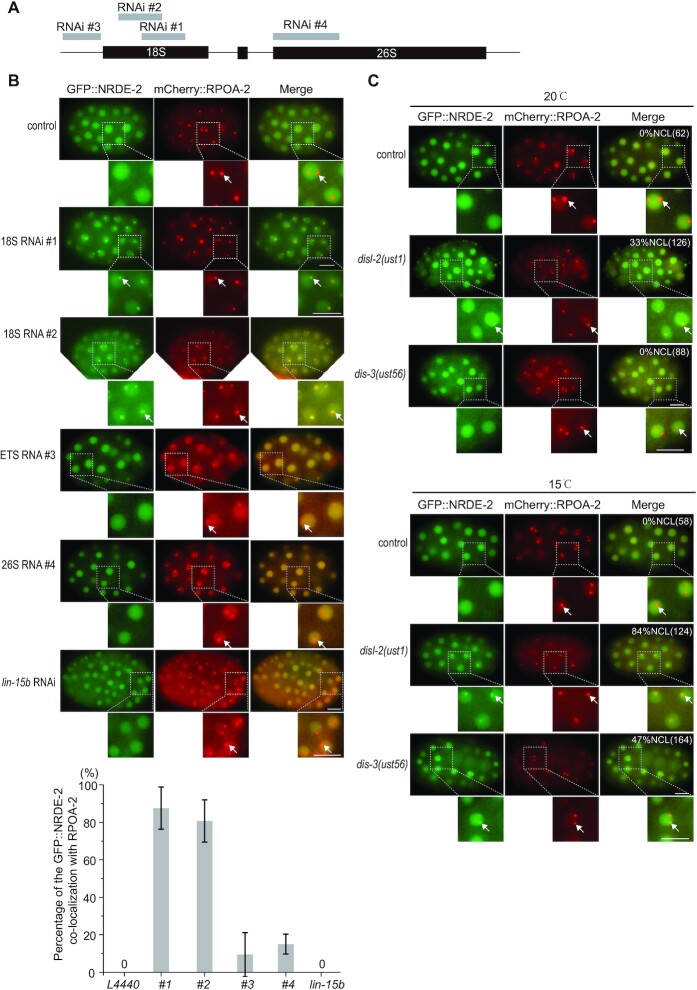
risiRNAs induced a nucleolar accumulation of NRDE-2. (**A**) Schematic of the dsRNAs targeting different regions of rDNA. (**B**) (top panel) Images of *C. elegans* embryos expressing GFP::NRDE-2 (green) and mCherry::RPOA-2 (red) after animals were fed with indicated dsRNA clones. *lin-15b* is a protein coding gene, which is used as a negative control. Scale bars, 10 μm. (botton panel) The percentage of embryos showing the colocalization of NRDE-2 with RPOA-2 were quantified. (**C**) Images of *C. elegans* embryos expressing GFP::NRDE-2 (green) and mCherry::RPOA-2 (red) in indicated animals grown at 20 and 15°C respectively. For the nucleolar NRDE-2 foci in embryos at 15°C in *disl-2* and *dis-3* mutants, worms were cultured at 15°C from L3 stage to adult stage, then embryos were photographed. Numbers indicate the percentages of the animals with nucleolar-enriched NRDE-2 in embryos (%NCL). The number of scored animals is indicated in parentheses.

To investigate the mechanism of nucleolar RNAi, we first tested a number of dsRNA clones targeting different rRNA sequences and found that dsRNA clones targeting 18S #1 and #2 were most effectively to induce the colocalization of NRDE-2 with RPOA-2 in the nucleoli (Figure 5A and B). However, feeding RNAi targeting 18S rRNA #1 and #2 did not significantly alter the brood size of animals (Supplementary Figure S7A). In *dis-3* and *disl-2* mutants, NRDE-2 also enriched in nucleoli and colocalized with RPOA-2 (Figure 5C), suggesting that both endogenous and exogenous source-derived risiRNAs may use a similar NRDE-dependent mechanism to silence rRNAs.

**Figure 6.**
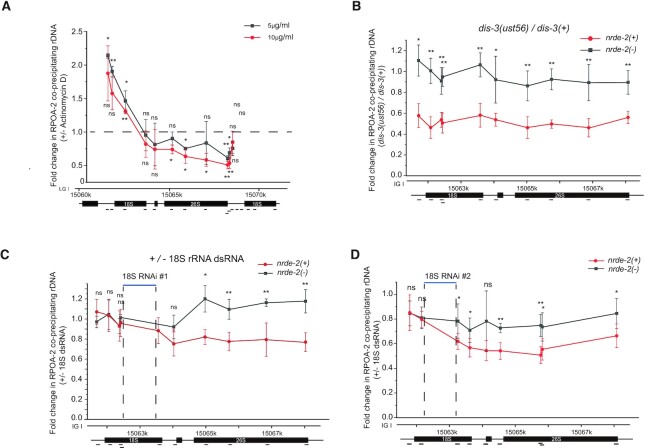
risiRNAs directed a NRDE-dependent inhibition of RNAP I transcription. (**A**) ChIP assay of RPOA-2 occupancy upon actinomycin D treatment. Fold changes were normalized to 1% input first, and then compared to the no-drug treatment group. Mean ± s.d.; *n* = 4; *P< 0.05 and **P < 0.01. (**B**) Results of the ChIP assay of RPOA-2 occupancy in the indicated animals. The enrichment of each sample was first normalized to 1% input. And then fold changes were calculated by dividing the enrichment of *dis-3(ust56)* mutants by the number of control animals. Statistics were performed by comparing the data from the *nrde-2(+)* and *nrde-2(–)* animals. mean ± s.d.; *n* = 4; *P< 0.05, **P < 0.01. (**C**, **D**) RPOA-2 occupancy along the rDNA unit was quantified by ChIP-qPCR upon RNAi targeting of 18S rRNA regions to indicated animals. The 18S RNAi #1 and #2 targeting region on the rDNA locis were shown in Figure 4A. Statistics were performed by comparing the data obtained for the *nrde-2(+)* and *nrde-2(–)* animals; mean ± s.d.; *n* = 5; *P < 0.05 and ** *P* < 0.01.

To determine how risiRNAs silence rRNA expression, we firstly assayed whether the GFP::RPOA-2 could recapitulate the function of endogenous proteins by ChIP assay. ChIP assay revealed that GFP::RPOA-2 could specifically bind to 18S, 5.8S and 26S rDNA, but not to 5S rDNA, which is consistent with the function of RNAP I in rRNA transcription (Supplementary Figure S7B). When animals were treated with actinomycin D, RPOA-2 paused at the 5′-end of the rRNA transcription unit and failed to elongate toward the 3′-end (Figure 6A), suggesting that the GFP::RPOA-2 fusion protein recapitulated the function of endogenous proteins.

We then investigated whether risiRNAs silences rRNA expression by inhibiting RNAP I transcription elongation. We quantified GFP::RPOA-2 occupancy by ChIP assay of the control animals, *dis-3* mutants, and animals being treated with RNAi targeting a fragment of 18S rRNAs. In *dis-3(ust56)* mutants, GFP::RPOA-2-associated rDNA was pronouncedly decreased, a phenomenon depending on the nuclear RNAi factor NRDE-2 (Figure 6B). In the absence of *nrde-2*, no change in RPOA-2 occupancy was observed. The mutation of *dis-3* or *nrde-2* did not significantly change the protein levels of GFP::RPOA-2 (Supplementary Figure S6D). Treating animals with exogenous dsRNA targeting a protein coding gene *oma-1* had no significant effect on RPOA-2 occupancy along the rRNA locus (Supplementary Figure S7C). Although treating animals with exogenous dsRNA targeting 18S rRNA had no significant effect on RPOA-2 occupancy near the site of transcription initiation and upstream of the RNAi-targeted site (Figure 6C and D), we detected a decrease in RPOA-2 occupancy downstream of the RNAi-targeted region. In addition, in the absence of *nrde-2*, risiRNAs failed to reduce RPOA-2 occupancy downstream of the RNAi-targeted region. Similar inhibition on transcription elongation had been observed for RNA polymerase II transcripts during nuclear RNAi targeting protein coding genes (18). Feeding RNAi with 26S dsRNA clone #4 had no significant effect on RPOA-2 occupancy (Supplementary Figure S7D).

Taken together, these data suggest that risiRNAs, acting together with the NRDE machinery, silence nascent RNAP I transcripts during the elongation phase of transcription in *C. elegans*.

### The proper nucleolar localization of exosomes was important for risiRNA suppression

**Figure 7.**
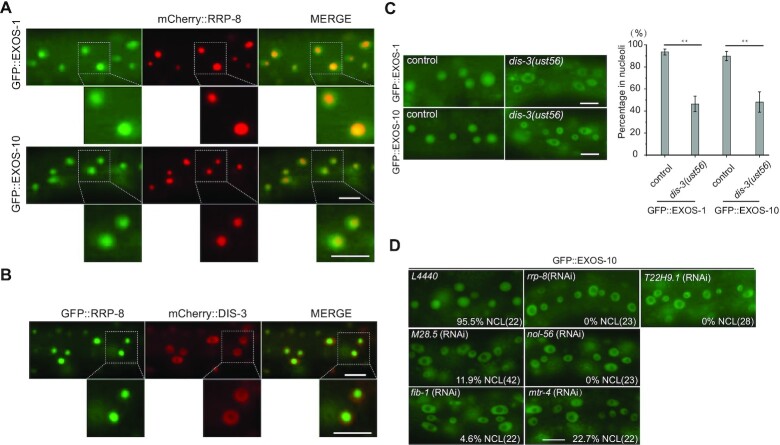
Candidate-based RNAi screening to search for factors that are required for the nucleolar localization of GFP::EXOS-10. (**A**) Images show somatic cells of L4 stage animals expressing GFP::EXOS-1 (green), GFP::EXOS-10 (green) and mCherry::RRP-8 (red). Scale bars, 10 μm. (**B**) Images show somatic cells of the L4 stage animals expressing GFP::RRP-8 (green) and mCherry::DIS-3 (red). Scale bars, 10 μm. (**C**) (left) Images of somatic cells of L4 stage animals. Scale bars, 10 μm. (right) Quantification of the nucleolar localization of GFP::EXOS-1 and GFP::EXOS-10. mean ± s.d.; *n* > 70 animals; **P < 0.01. (**D**) Images of somatic cells of L4 animals expressing GFP::EXOS-10 after being treated with RNAi targeting the indicated genes. Bleached embryos carrying exosome transgenes were grown on RNAi plates to L4 stage for photographing. The percentage of animals with nucleolar localized GFP::EXOS-10 is indicated (% NCL). The number of scored animals is indicated in parentheses. Scale bars, 10 μm. Animals were cultured at 20°C.

To further investigate the biological roles of the exosome complex in risiRNA production in *C. elegans*, we constructed fluorescent protein-tagged exosome subunits, including mCherry::DIS-3, GFP::EXOS-1, GFP::EXOS-2 and GFP::EXOS-10. These subunits are ubiquitously expressed in all of the cells and enriched in the nucleus (Supplementary Figure S8A–C). We also constructed mCherry- and GFP-tagged RRP-8, which exclusively localized in the nucleolus. We crossed GFP::EXOS-1 and GFP::EXOS-10 onto a mCherry::RRP-8 background, respectively, and found that EXOS-1 and EXOS-10 were enriched in nucleoli and colocalized with RRP-8 in somatic cells (Figure 7A and Supplementary Figure S9A). After crossing mCherry::DIS-3 with GFP::RRP-8 animals, we found that DIS-3 was enriched in the nucleoplasm but depleted from nucleoli (Figure 7B and Supplementary Figure S9B). Then we crossed GFP::EXOS-1 and GFP::EXOS-10 onto the *dis-3(ust56)* mutant. Surprisingly, EXOS-1 and EXOS-10 were depleted from the nucleoli but enriched in the nucleoplasm (Figure 7C). Similar phenomena were observed in human cells (37). Both feeding RNAi targeting 18S rRNA and mutation of *disl-2* did not induce the mislocalization of GFP::EXOS-10 from the nucleoli to nucleoplasm (Supplementary Figure S9C and D).

To demonstrate that the proper nucleolar localization of the exosome complex is important for risiRNA suppressing, we performed a candidate-based RNAi screening to search for rRNA processing factors that are required for the nucleolar localization of GFP::EXOS-10. We selected fifteen predicted rRNA processing factors and investigated whether knocking down these genes by RNAi could block the nucleolar accumulation of EXOS-10 (Supplementary Table S1). We found that knocking down *M28.5*, *nol-56*, *fib-1* and *mtr-4* by RNAi induced a dramatic depletion of EXOS-10 from the nucleoli (Figure 7D). Among the proteins encoded by these genes, RRP-8 and T22H9.1 are known SUSI proteins that are involved in the modification and processing of rRNAs (14). Knocking down *rrp-8* and *T22H9.1* by RNAi induced the depletion of GFP::EXOS-10 from the nucleoli (Figure 7D) and the increase of risiRNAs (14).

**Figure 8.**
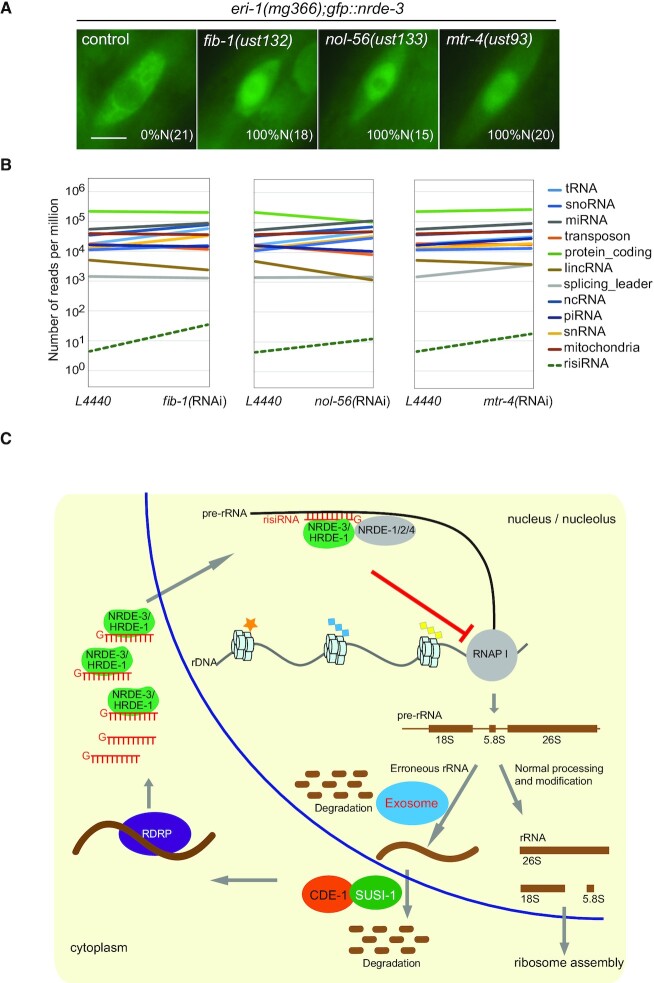
risiRNAs were enriched in the mutants of rRNA processing and maturation factors. (**A**) Images of seam cells from L2 stage animals. Numbers indicate the percentages of the animals with nuclear-enriched NRDE-3 in seam cells (%N). The number of scored animals is indicated in parentheses. Scale bars, 5 μm. (**B**) Results from the deep sequencing of total small RNAs from the indicated animals. (**C**) A working model of risiRNA biogenesis and function. The processes of ribosome biogenesis are very sophisticated in eukaryotic cells from the splicing events of pre-rRNAs to the final assemblage of ribosomes, during which errors could occur at any step. In the nucleoli and nucleus, an exoribonucleolytic multisubunits protein complex, the exosome, participates in rRNA processing and intermediates degradation. In the cytoplasm, erroneous rRNAs are uridylated at the 3′-ends by polyuridylating polymerase-I (named CDE-1 or PUP-1) and then degraded by the 3′ to 5′ exoribonuclease DISL-2. Deficiency of these two degradation systems results in the accumulation of erroneous uridylated rRNAs, which further recruit additional RNA-dependent RNA polymerases (RdRPs) to synthesize risiRNAs and initiate the nucleolar gene silencing cascade. risiRNAs associate with the nuclear Argonaute protein NRDE-3 in soma or HRDE-1 in the germline, bind to pre-rRNAs, and inhibit RNAP I transcription elongation. Therefore, by combining the RNA degradation system with nucleolar gene silencing machinery, cells surveil the quality of rRNAs and maintain the rRNA homeostasis.

NOL-56 is an ortholog of human NOP56, which binds snoRNAs and facilitates box C/D ribonucleoprotein-guided methyltransferase activity (38). FIB-1 encodes the *C. elegans* ortholog of human fibrillarin and *Saccharomyces cerevisiae* Nop1p. FIB-1 has RNA binding and rRNA methyltransferase activities, which are essential for nucleologenesis (39). MTR-4 is an ortholog of human MTREX and has ATP-dependent RNA helicase activity (28). We deleted *fib-1*, *nol-56* and *mtr-4* by CRISPR/Cas9 technology (Supplementary Figure S10A). In the mutants, NRDE-3 redistributed from the cytoplasm to the nucleus in seam cells (Figure 8A). In addition, after knocking down these genes by RNAi, risiRNAs were modestly enriched, as shown by total small RNA deep sequencing (Figure 8B).

To test the specificity of nucleolus localization of exosome components, we examined the nucleolar localization of RBD-1, FIB-1 and RRP-8 upon knockdown exosome components and factors identified in Figure 7. The change of nucleolar localization of the three proteins were not detectable (Supplementary Figure S11), suggesting that the integrity of nucleoli was maintained. The depletion of M28.5 might hinder FIB-1 assembly into the box C/D complex then cause an abnormal localization of FIB-1 proteins (Supplementary Figure S11B).

These data suggested that proper nucleolar localization of the exosome complex was important for the suppression of risiRNA production, and can be used as a tool to search for new *susi* genes. Yet a direct causative relationship between exosome mislocalization and risiRNA production remains to be determined.

## DISCUSSION

Eukaryotic cells express a multitude of small regulatory RNAs and antisense transcripts that are of unknown function (40). Small RNAs have been shown to induce endonucleolytic cleavage of target RNAs (slicer activity) or induce epigenetic modifications, including DNA and histone modifications (41). In *C. elegans*, the nuclear Argonaute protein NRDE-3 in soma (or HRDE-1 in the germline) lacks the residues required for slicer activity but inhibits RNAP II-mediated transcription elongation in the presence of siRNAs (18). Here, we showed that risiRNAs guide the NRDE complex to pre-rRNAs to inhibit RNAP I transcription (Figure 8C). Thus, our data suggest a mechanism for nucleolar RNAi: risiRNA-directed cotranscriptional silencing of RNAP I. NRDE-2 is a conserved protein, which is involved in the processing of pre-mRNAs in mammalian cells (42–44). It will be of interest to investigate whether risiRNAs and RNAP I are similarly linked in other metazoans (12).

We failed to detect significant change of H3K9 and H3K27 trimethylation at the rDNA locus in the presence of risiRNA. Small-RNA-guided chromatin modifications have been widely studied in many organisms. In *C. elegan*s, NRDE complex transports 22G RNAs from the cytoplasm to the nucleus, induces H3K9, H3K23 and H3K27 trimethylation and mediates transgenerational inheritance of RNAi (34,45). In this study, we found that the risiRNA/NRDE complex inhibits RNAP I transcription without significantly altering the status of H3K9 and H3K27 trimethylation of rDNA genes. However, it is unclear whether risiRNA-induced nucleolar RNAi is independent of H3K9 and H3K27 trimethylation or not. rDNA is a multicopy gene, approximately 50 copies in *C. elegans*, while only a proportion of the copies are actively transcribed in many organisms (46–49). The signal to noise for the rDNA loci by ChIP assay could potentially be worse than that for the single-copy protein coding genes if the histone modification changes only occurred at a few copies. In addition, each copy may not be equally affected by risiRNA-mediated nucleolar RNAi. In *S. cerevisiae*, actively transcribed rDNA genes are largely devoid of histone molecules and are organized in a specialized chromatin structure that binds the high-mobility group protein Hmo1 (50). Reducing the rDNA transcription efficiency upon the depletion of *dao-5* does not induce significant change of H3K9me3 modification at rDNA region in *C. elegans* (51), supporting the idea that inhibiting RNAP I transcription may not rely on prior H3K9 and H3K27 methylation. Further study to identify which types of histone modifications are engaged in rDNA silencing will facilitate the understanding of the mechanism and regulation of risiRNA-directed RNAP I inhibition.

Although the addition of external dsRNA and production of risiRNAs upon loss of exosome components exhibit sequence complementary to different regions of rRNAs, risiRNAs generated under two conditions are quite similar. Three lines of evidence cumulatively support the idea. First, both conditions elicited the nuclear and nucleolar accumulation of NRDE-2 and NRDE-3. Second, they induced NRDE-2 and NRDE-3 association with pre-rRNAs (Figures 4 and 5). Third, the majority of both types of NRDE-3-associated antisense risiRNAs were 22G RNAs and depended on RNA-dependent RNA polymerases. Therefore, although different rRNA errors may involve the risiRNA production machinery at distinct steps, they may use a similar NRDE-dependent pathway to silence pre-rRNAs.

We have tested a number of dsRNA clones to induce the nucleolar localization of NRDE-2 (Figure 5A and B). For unknown reasons, we found that only a few RNAi clones induced the strongest effect on nucleolar localization of the NRDE proteins. We selected RNAi clone to target a region of 26S rRNA and failed to detect a strong nucleolar accumulation of NRDE-2 (Figure 5A and B) and decrease of RPOA-2 (Supplementary Figure S7D). We do not know why some dsRNAs are more efficient to induce nuclear or nucleolar RNAi. It is possible that the high-order structure in distinct RNA regions may determine the amplification efficiency of secondary siRNAs by RNA-dependent RNA polymerases or affect the binding of siRNA/NRDE complex with nascent transcripts.

The translocation of exosome factors is very intriguing. We speculate that the exosome translocation may respond to the accumulation of erroneous rRNAs, but not directly requires or induces risiRNA production. The relocalization of EXOSC10 has been observed in human cells depleted for DIS3 (37) and been attributed to sequestration of nucleolar exosome factors to the nucleoplasm in cases where nucleoplasmic exosome activity is limiting. We tried hard but failed to directly sequester exosome components into distinct subnuclear compartments by tagging them with different localization signals. Therefore, we do not know whether exosome translocation *per se* is the cause or the outcome of risiRNA generation. However, in *disl-2* mutant, the subnuclear localization of exosome component EXOS-10 is unchanged (Supplementary Figure S9D). Yet in *disl-2* mutant, risiRNAs elevated (5). *DISL-2(SUSI-1)* is a cytoplasmic localized SUSI protein that degrades erroneous rRNA from 3′ to 5′ end. Therefore abnormal exosome function is not a common mechanism for increased risiRNA production in all *susi(-)* conditions.

The biological roles the nucleolar RNAi pathway is still unclear. We did find risiRNAs inhibit the expression of rRNAs, slow down the growth of animals and reduced the brood size (5,14). One possibility is that rRNA is so essential for organisms and its quality and quantity will be strictly scrutinized. When there is a genetic mutation that alter the quality and quantity of rRNAs, organism will find a way to quickly eliminate the mutants from the population, to keep the safety of the species, other than keep the poisonous mutation. Alternatively, this mechanism may be beneficial for animals to respond to certain environmental challenges.

The processing of ribosomal RNAs is extraordinarily complicated and defects may occur at every step from production to assembly and cause ribosomopathies (1,52). Multiple surveillance machineries, including the nuclear-localized RNA exosome complex and the cytoplasmic exoribonuclease DISL-2, degrade defective rRNAs (3,28,53,54). The deficiencies of surveillance machinery result in the accumulation of erroneous rRNAs that could be harmful for the cell metabolism. However, *C. elegans* utilizes a backup system, nucleolar RNAi, in which risiRNAs are produced to induce a nucleolar gene silencing by inhibiting RNAP I transcription. Therefore, these two systems act together to maintain rRNA homeostasis and prohibit the accumulation of erroneous rRNAs.

## DATA AVAILABILITY

All raw and normalized sequencing data have been deposited to Gene Expression Omnibus under submission number GSE165078.

## Supplementary Material

gkab662_Supplemental_File
