# Supplementary material for: Antisense ribosomal siRNAs inhibit RNA polymerase I-directed transcription in C. elegans
Source: Nucleic Acids Res. 2021 Aug 7;49(16):9194–210. doi: 10.1093/nar/gkab662 (PMC8450093; doi:10.1093/nar/gkab662)
Supplement: gkab662_Supplemental_File [file gkab662_supplemental_file.pdf]

Supplementary figure legends:

Fig. S1. *susi-5* was identified as *dis-3*. (A) Schematic of SUSI-5(ceDIS-3) (top) and sequence alignments of DIS-3 in different organisms: hs, *homo sapiens*; mm, *Mus musculus*; sp, *Schizosaccharomyces pombe*; dm, *Drosophila melanogaster*; and ce, *C. elegans*. (B) Images of seam cells of *eri-1(mg366);dis-3(ust56);gfp::nrde-3;mCherry::dis-3* animals at L4 stage. mCherry::DIS-3 rescued the cytoplasmic localization of GFP::NRDE-3 in *eri-1(-)* animals. Scale bars, 10 µm. (C) Brood size of the *dis-3* mutant grown at 20°C and 25°C, respectively. mean ± s.d.; n > 10 animals.

Fig. S2. risiRNAs were accumulated in *dis-3(ust56)* mutants. (A) Results from the deep sequencing of total small RNAs from indicated animals. Green dashed lines indicate risiRNAs. (B) Distribution from the deep sequencing of NRDE-3-associated small RNAs in indicated animals. Sense fragments of lincRNA, protein-coding transcripts, transposon transcripts, ncRNA and ribosomal RNA are not counted. (C) Results from the deep sequencing of total small RNAs from the indicated animals. Green dashed lines indicate risiRNAs.

Fig. S3. Alleles of the exosome subunits used in this study. (A) Brood sizes of indicated animals grown at 20°C. Data are presented as mean ± s.d.; n > 10 animals. Progeny reached L4 or young adult stages are counted to evaluate the fertility. (B-J) The *ust* alleles were generated via a dual sgRNA-directed CRISPR/Cas9 gene knockout technology in *C. elegans.* The *tm* alleles and *ok* alleles were acquired from National Bioresource Project and the CGC, respectively. *exos-5(ust61), exos-7(ust62),* and *exos-10(ok2269)* alleles are in-frame mutations and the translated products are predicted to lose 120, 89 and 208 amino acids respectively. All other alleles are frame-shift mutations, and likely nulls. Red bars indicate deleted regions in gene loci. Green arrows indicate premature stop codons.

Fig. S4. RPOA-2 localized to nucleoli in embryos. (A) Brood size of wild type animals upon RNAi targeting of *rpoa-2*. Data are presented as mean ± s.d.; n=16 animals. (B)

Images of young gravid adult animals expressing GFP::RPOA-2. Scale bars, 25 μm. (C) Images of *C. elegans* embryos expressing GFP:RPOA-2 (green) and mCherry::FIB-1 (red). Scale bars, 10 μm. (D) Images of the embryos expressing GFP::RPOA-2. Scale bars, 25 μm. White arrows indicate nucleoli. Scale bars, 10 μm.

Fig. S5. Images of the animals expressing indicated transgenes in the presence of actinomycin D. Shown are embryos (A), soma of L4 stage animals (B), and germline (C). White arrows indicate nucleoli. Scale bars, 10 μm.

Fig. S6. *dis-3* mutation did not change the expression of RPOA-2. (A) Images of seam cells of L4 stage animals expressing GFP::NRDE-3 in the presence of actinomycin D in the *eri-1(mg366)* background. Scale bars, 5 μm. (B) Images show somatic cells of L4 stage animals expressing GFP::EXOS-10 in the presence of actinomycin D. The percentage of animals with nucleolar localized GFP::EXOS-10 is indicated (% NCL). The number of scored animals is indicated in parentheses. Scale bars, 10 μm. (C) Western Blotting of GFP::RPOA-2 in indicated actinomycin D-treated animals. (D) Western Blotting of GFP::RPOA-2 in indicated animals.

Fig. S7. (A) Feeding RNAi targeting 18S rRNAs did not significantly change the brood size of animals. Animals were grown at 20ºC. Data are presented as mean ± s.d; n > 10 animals; ns, nonsignificance. (B) Results of the ChIP assay of RPOA-2 occupancy at rDNA loci by non-specific IgG or anti-GFP antibody. The enrichment of each sample was normalized to 1% input. Statistics were performed by comparing the data from the IgG or anti-GFP antibody groups. Mean ± s.d.; n = 4, ***P<0.001. (C) Results of the ChIP assay of RPOA-2 at rDNA loci after feeding dsRNA targeting a protein coding gene *oma-1*. (D) Results of the ChIP assay of RPOA-2 at rDNA loci after feeding dsRNA targeting a 26S rRNA segment. The enrichment of each sample was first normalized to 1% input. And then fold changes were calculated by dividing the enrichment of indicated mutants by the number of control animals.

Fig. S8. Images of the animals expressing indicated transgenes. (A) L3 animals and (B) germline of young gravid adults. Scale bars, 25 μm. (C) Images of 1-to-8 cell embryos from the animals expressing indicated transgenes. Scale bars, 10 μm.

Fig. S9. Subcellular localization of the exosome subunits of L4 stage animals. (A) Images of somatic cells of the animals expressing indicated transgenes. The fluorescence intensity indicated by dashed lines was measured by ImageJ, and the values are shown on the right. Scale bars, 5 μm. (B) Images of somatic cells of the animals expressing indicated transgenes. Scale bars, 5 μm. (C) (left) Images of somatic cells of the indicated animals. Scale bars, 10 μm. (right) Quantification of the nucleolar localization of GFP::EXOS-10. mean ± s.d.; n > 70 animals; ns, not significant. (D) (top) Images of somatic cells of the indicated animals. Scale bars, 10 μm. (bottom) Quantification of the nucleolar localization of GFP::EXOS-10. mean ± s.d.; n > 70 animals; ns, not significant.

Fig. S10. Schematics of the alleles that were generated via dual sgRNA-directed CRISPR/Cas9 gene knockout technology. Each of the alleles contains a frameshift mutation and is likely a null allele.

Fig. S11. The localization of nucleolar proteins. The expression and localization of mCherry::RBD-1 (A), mCherry::FIB-1 (B) and GFP::RRP-8 (C) upon RNAi indicated genes. Bleached embryos were placed on RNAi plates and grew up to L4 stage for photographing. Scale bars, 10 μm.

Table S1. Candidate-base RNAi screening for factors affecting the subcellular localization of GFP::EXOS-10. The percentage of animals with nucleolar-localized GFP::EXOS-10 is indicated (% NCL).

Table S2. List of strains used in this study.

Table S3. Sequences of sgRNAs for CRISPR/Cas9-mediated gene editing.

Table S4. Sequences of quantitative real-time PCR primers for ChIP experiments.

Table S5. Sequences of quantitative real-time PCR primers for detecting the pre-rRNA and rRNA levels.

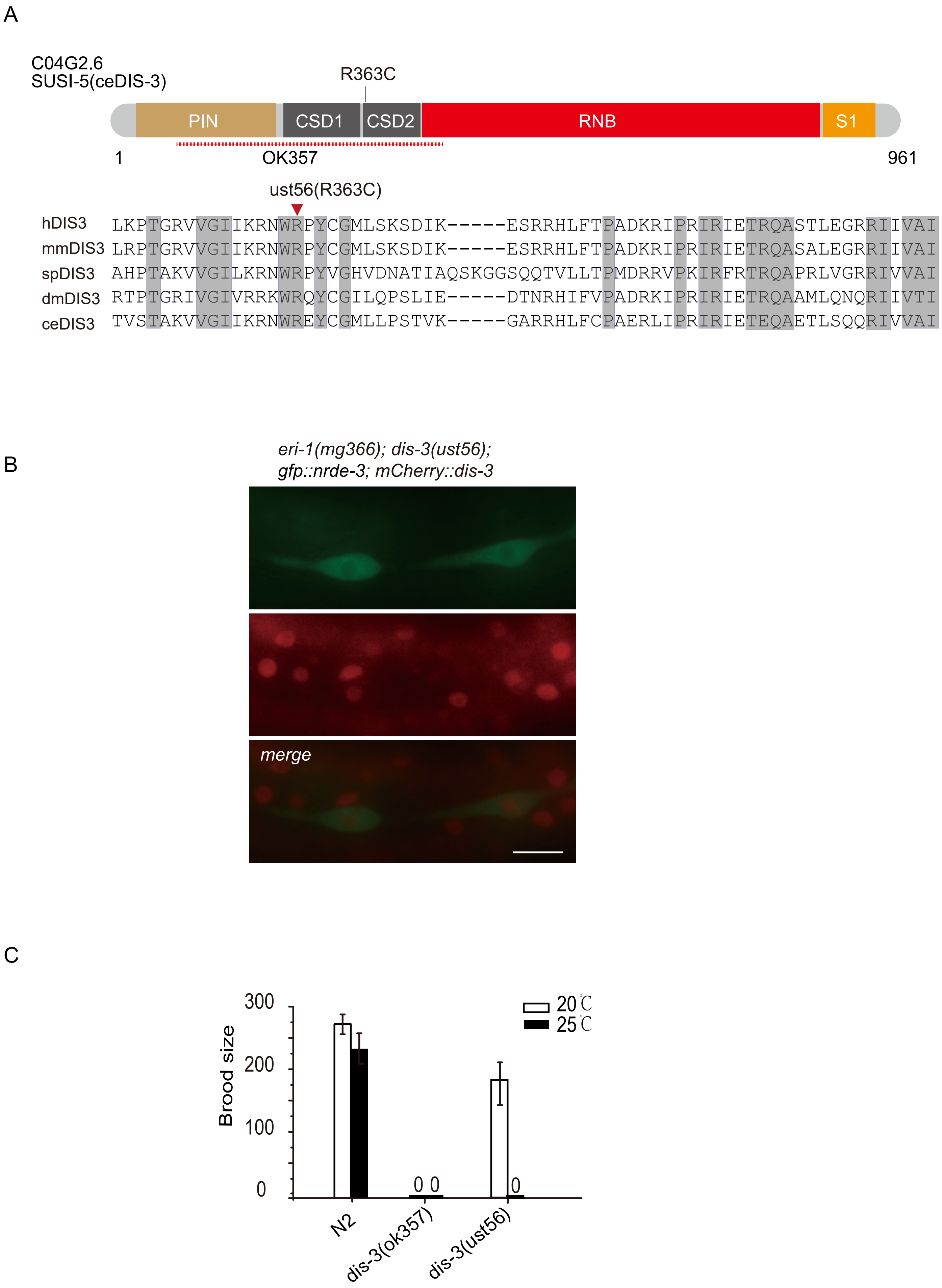
A
C04G2.6
SUSI-5(ceDIS-3)
R363C
PIN
CSD1
CSD2
RNB
S1
1
OK357
961
ust56(R363C)
hDIS3 LKPTGRVVGIIKRNWRPYCGMLSKSDIK-----ESRRHLFTPADKRIPRIRIETRQASTLEGRRIIVAI
mmDIS3 LRPTGRVVGIIKRNWRPYCGMLSKSDIK-----ESRRHLFTPADKRIPRIRIETRQASALEGRRIIVAI
spDIS3 AHPTAKVVGILKRNWRPYVGHVDNATIAQSKGGSQQTVLLTPMDRRVPKIRFRTRQAPRLVGRRIVVAI
dmDIS3 RTPTGRIVGIVRRKWRQYCGILQPSLIE-----DTNRHIFVPADRKIPRIRIETRQAAMLQNQRIIVTI
ceDIS3 TVSTAKVVGIIKRNWREYCGMLLPSTVK-----GARRHLFCPAERLIPRIRIETEQAETLSQQRIVVAI
B
eri-1(mg366); dis-3(ust56);
gfp::nrde-3; mCherry::dis-3
merge
C
Brood size
300
200
100
0
20℃
25℃
0 0
0
N2
dis-3(ok357)
dis-3(ust56)

Figure S1

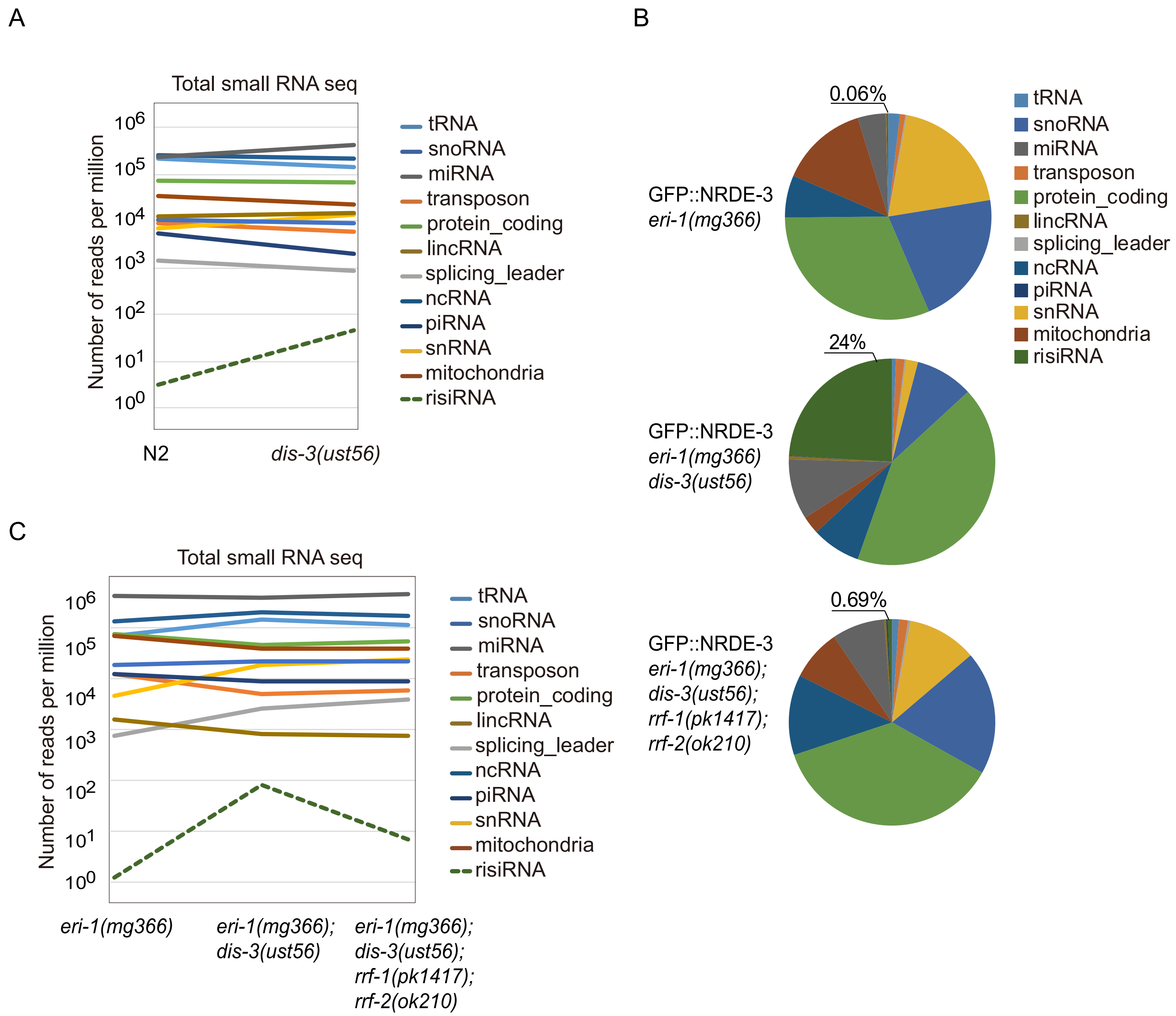
A
Total small RNA seq
Number of reads per million
10^6
10^5
10^4
10^3
10^2
10^1
10^0
N2
dis-3(ust56)
tRNA
snoRNA
miRNA
transposon
protein_coding
lincRNA
splicing_leader
ncRNA
piRNA
snRNA
mitochondria
risiRNA
B
0.06%
GFP::NRDE-3
eri-1(mg366)
24%
GFP::NRDE-3
eri-1(mg366)
dis-3(ust56)
0.69%
GFP::NRDE-3
eri-1(mg366);
dis-3(ust56);
rrf-1(pk1417);
rrf-2(ok210)
tRNA
snoRNA
miRNA
transposon
protein_coding
lincRNA
splicing_leader
ncRNA
piRNA
snRNA
mitochondria
risiRNA
C
Total small RNA seq
Number of reads per million
10^6
10^5
10^4
10^3
10^2
10^1
10^0
eri-1(mg366)
eri-1(mg366);
dis-3(ust56)
eri-1(mg366);
dis-3(ust56);
rrf-1(pk1417);
rrf-2(ok210)
tRNA
snoRNA
miRNA
transposon
protein_coding
lincRNA
splicing_leader
ncRNA
piRNA
snRNA
mitochondria
risiRNA

Figure S2

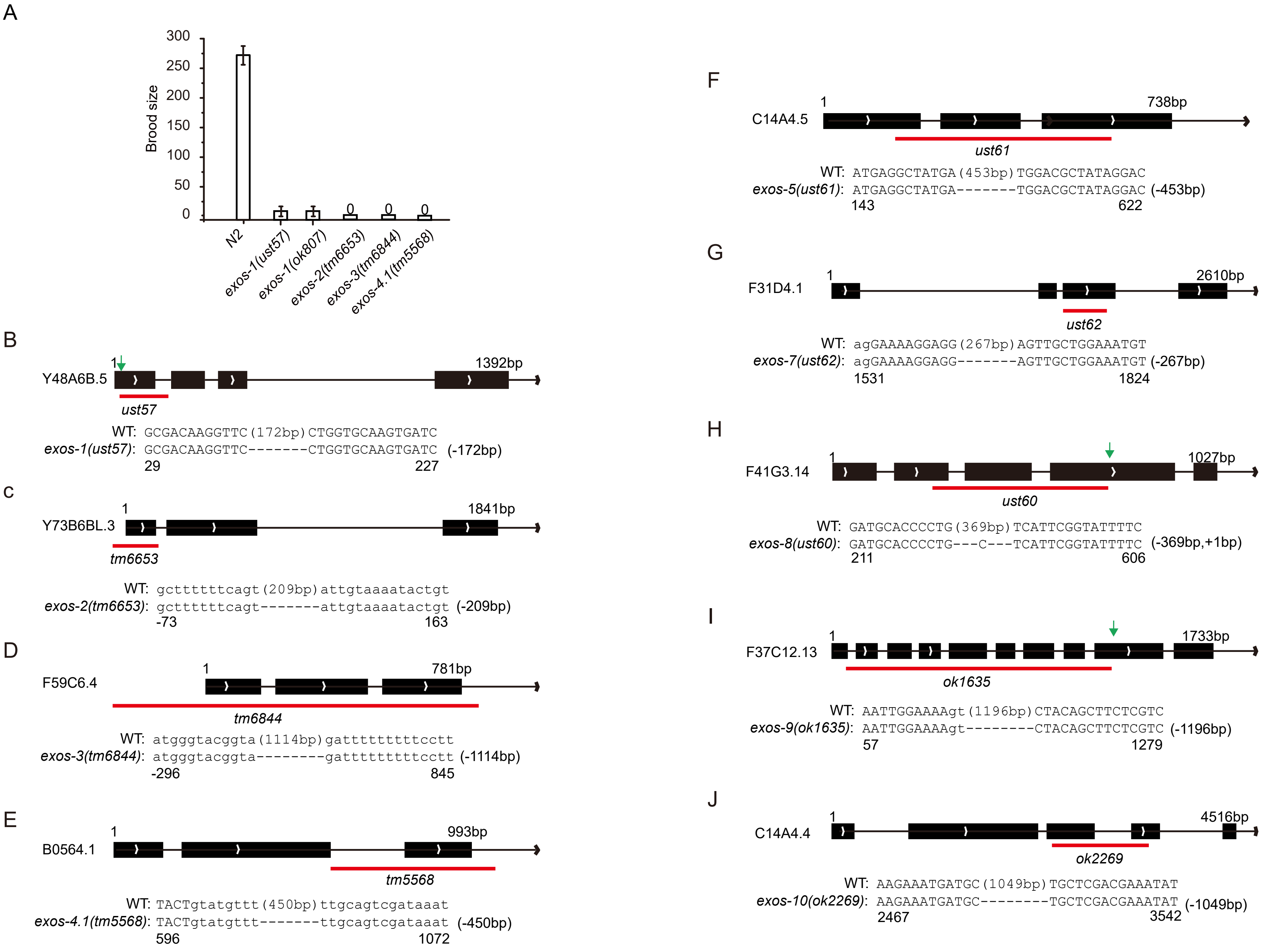
A
Brood size
300
250
200
150
100
50
0
0
0
0
N2
exos-1(ust57)
exos-1(ok807)
exos-2(tm6653)
exos-3(tm6844)
exos-4.1(tm5568)
B
Y48A6B.5
1
1392bp
ust57
WT: GCGACAAGGTTC(172bp)CTGGTGCAAGTGATC
exos-1(ust57): GCGACAAGGTTC-------CTGGTGCAAGTGATC (-172bp)
29
227
C
Y73B6BL.3
1
1841bp
tm6653
WT: gctttttttcagt(209bp)attgtaaaatactgt
exos-2(tm6653): gctttttttcagt-------attgtaaaatactgt (-209bp)
-73
163
D
F59C6.4
1
781bp
tm6844
WT: atgggtacggta(1114bp)gatttttttttcctt
exos-3(tm6844): atgggtacggta--------gatttttttttcctt (-1114bp)
-296
845
E
B0564.1
1
993bp
tm5568
WT: TACTgtatgttt(450bp)ttgcagtcgataaat
exos-4.1(tm5568): TACTgtatgttt-------ttgcagtcgataaat (-450bp)
596
1072
F
C14A4.5
1
738bp
ust61
WT: ATGAGGCTATGA(453bp)TGGACGCTATAGGAC
exos-5(ust61): ATGAGGCTATGA-------TGGACGCTATAGGAC (-453bp)
143
622
G
F31D4.1
1
2610bp
ust62
WT: agGAAAAGGAGG(267bp)AGTTGCTGGAAATGT
exos-7(ust62): agGAAAAGGAGG-------AGTTGCTGGAAATGT (-267bp)
1531
1824
H
F41G3.14
1
1027bp
ust60
WT: GATGCACCCCTG(369bp)TCATTCGGTATTTTC
exos-8(ust60): GATGCACCCCTG---C---TCATTCGGTATTTTC (-369bp,+1bp)
211
606
I
F37C12.13
1
1733bp
ok1635
WT: AATTGGAAAAgt(1196bp)CTACAGCTTCTCGTC
exos-9(ok1635): AATTGGAAAAgt--------CTACAGCTTCTCGTC (-1196bp)
57
1279
J
C14A4.4
1
4516bp
ok2269
WT: AAGAAATGATGC(1049bp)TGCTCGACGAAATAT
exos-10(ok2269): AAGAAATGATGC--------TGCTCGACGAAATAT (-1049bp)
2467
3542

Figure S3

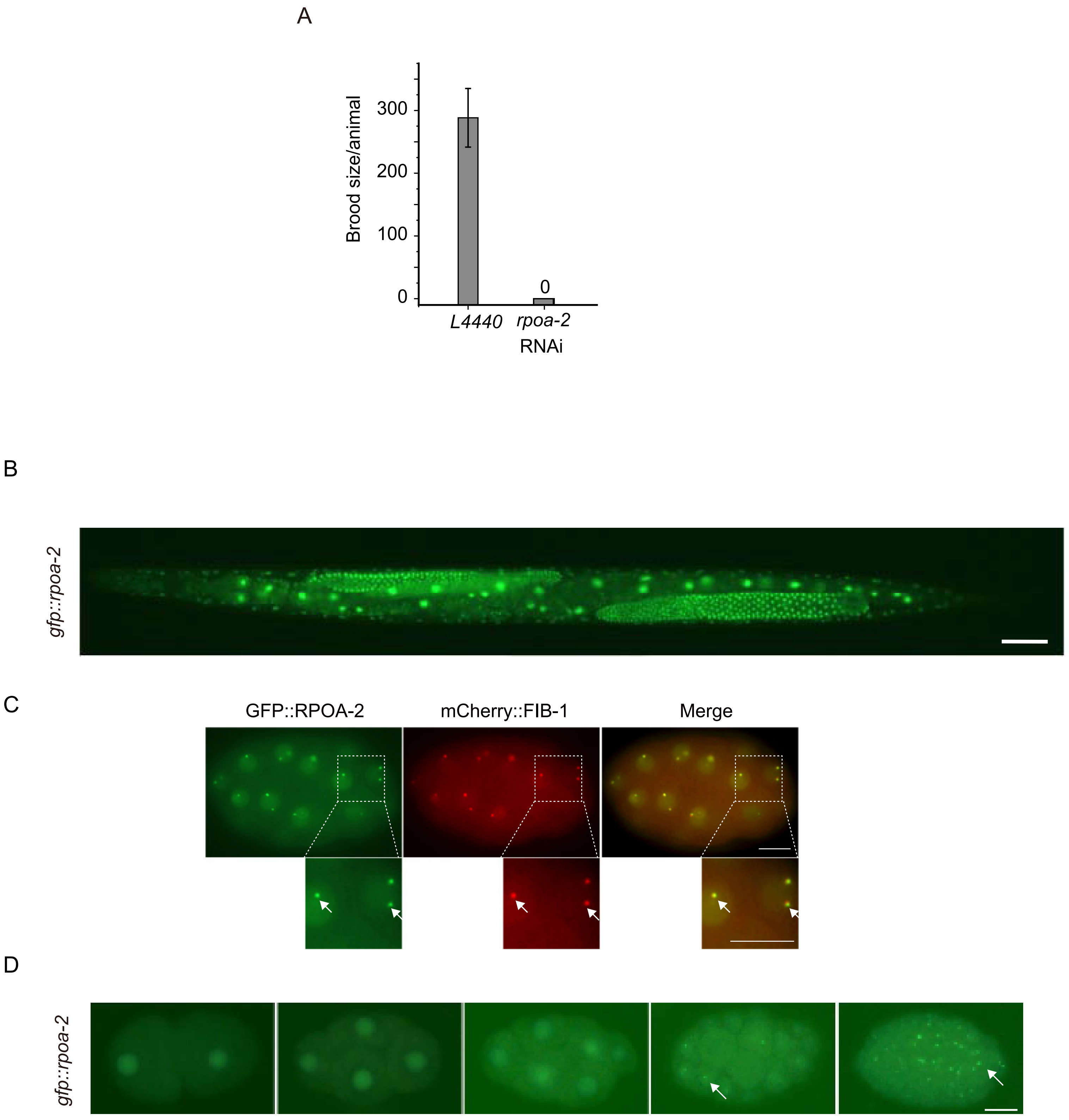
A
Brood size/animal
300
200
100
0
0
L4440
rpoa-2
RNAi
B
gfp::rpoa-2
C
GFP::RPOA-2
mCherry::FIB-1
Merge
D
gfp::rpoa-2

Figure S4

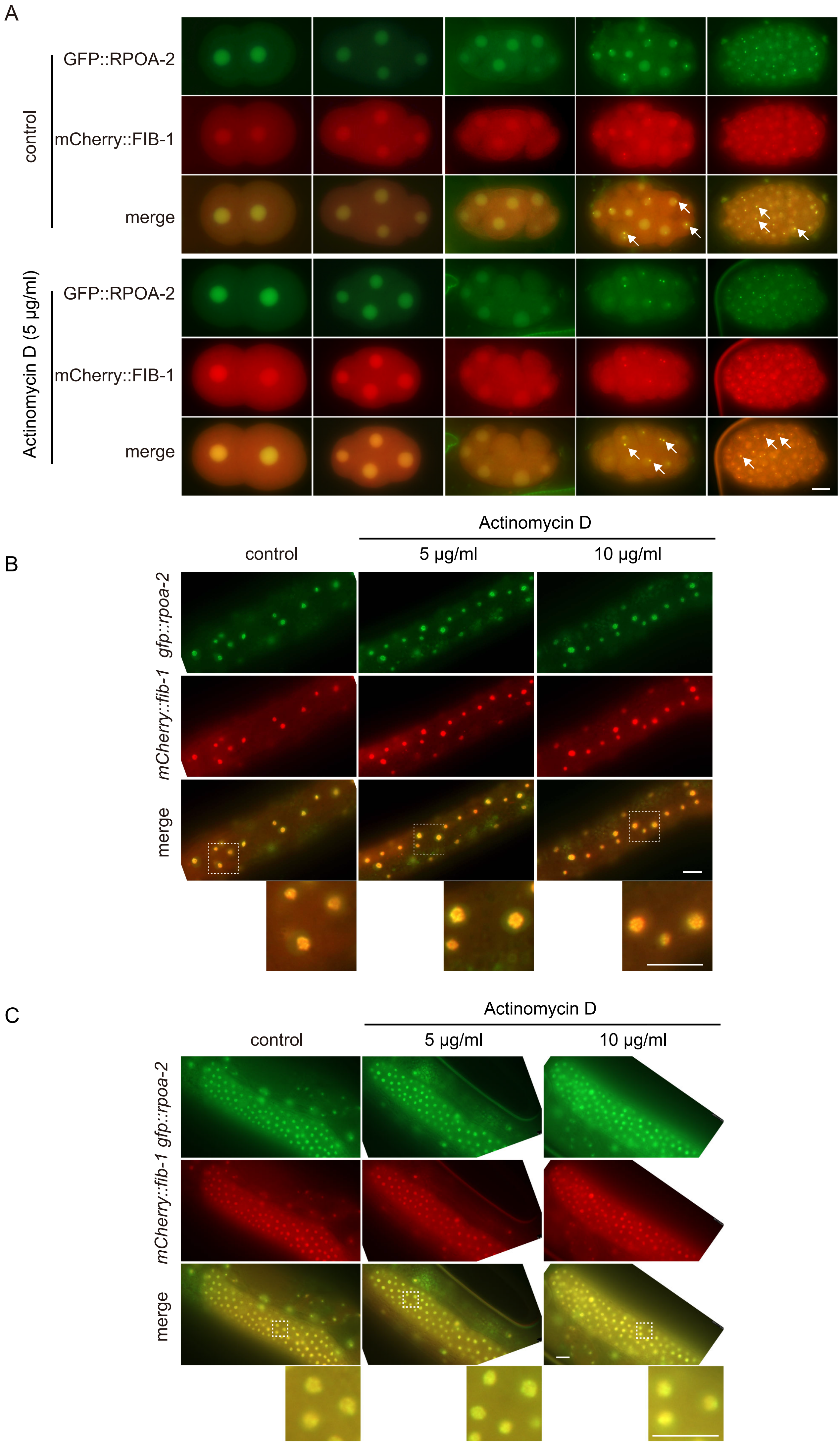
A
control
GFP::RPOA-2
mCherry::FIB-1
merge
Actinomycin D (5 µg/ml)
GFP::RPOA-2
mCherry::FIB-1
merge
B
Actinomycin D
control
5 µg/ml
10 µg/ml
gfp::rpoa-2
mCherry::fib-1
merge
C
Actinomycin D
control
5 µg/ml
10 µg/ml
gfp::rpoa-2
mCherry::fib-1
merge

Figure S5

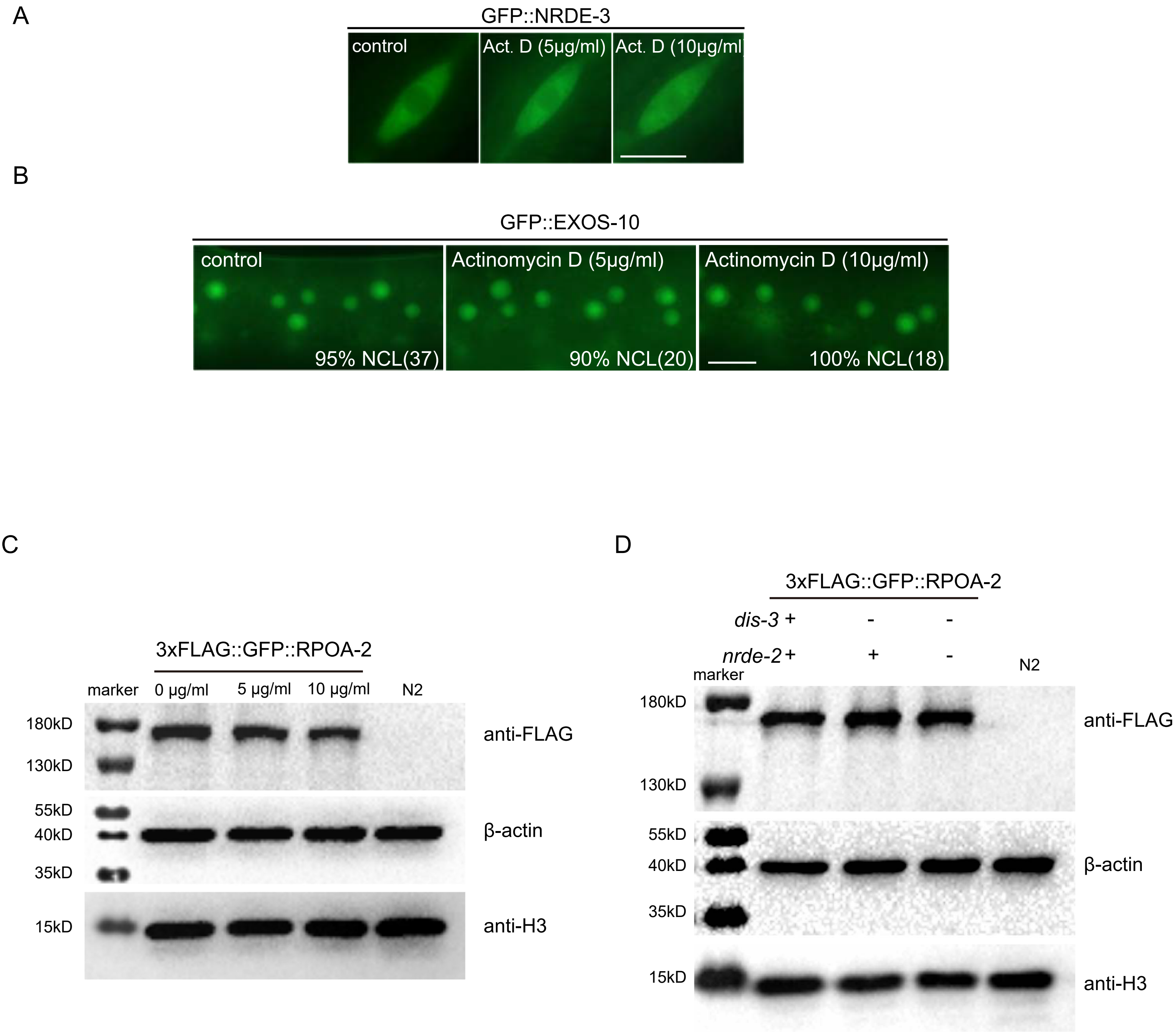
A
GFP::NRDE-3
control
Act. D (5μg/ml)
Act. D (10μg/ml
B
GFP::EXOS-10
control
Actinomycin D (5μg/ml)
Actinomycin D (10μg/ml)
95% NCL(37)
90% NCL(20)
100% NCL(18)
C
3xFLAG::GFP::RPOA-2
marker
0 μg/ml
5 μg/ml
10 μg/ml
N2
180kD
130kD
55kD
40kD
35kD
15kD
anti-FLAG
β-actin
anti-H3
D
3xFLAG::GFP::RPOA-2
dis-3 + - -
nrde-2 + + -
marker
N2
180kD
130kD
55kD
40kD
35kD
15kD
anti-FLAG
β-actin
anti-H3

Figure S6

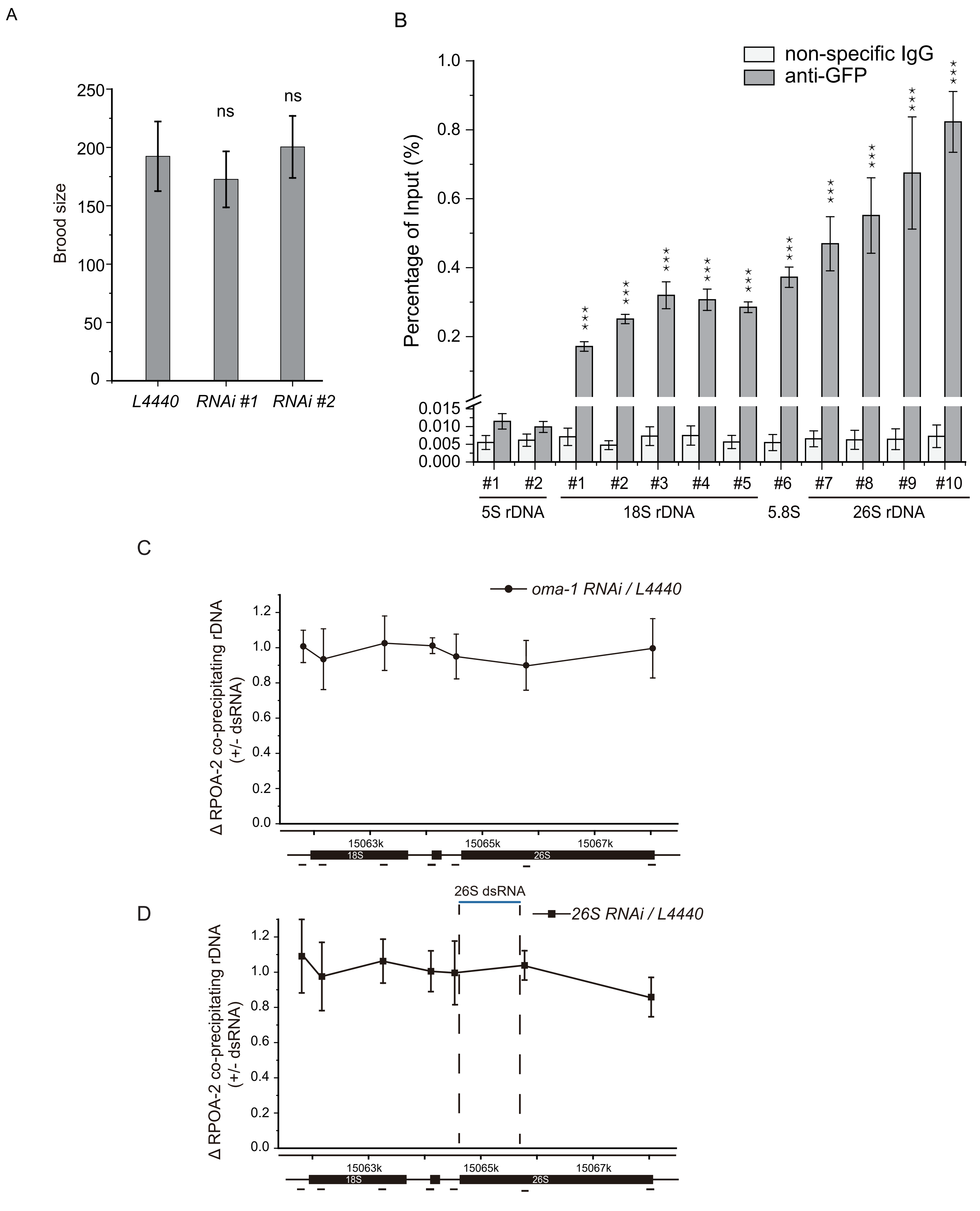
A
B
C
D
Brood size
ns
L4440
RNAi #1
RNAi #2
non-specific IgG
anti-GFP
Percentage of Input (%)
5S rDNA
18S rDNA
5.8S
26S rDNA
oma-1 RNAi / L4440
26S dsRNA
26S RNAi / L4440
Δ RPOA-2 co-precipitating rDNA (+/- dsRNA)
15063k
15065k
15067k
18S
26S

Figure S7

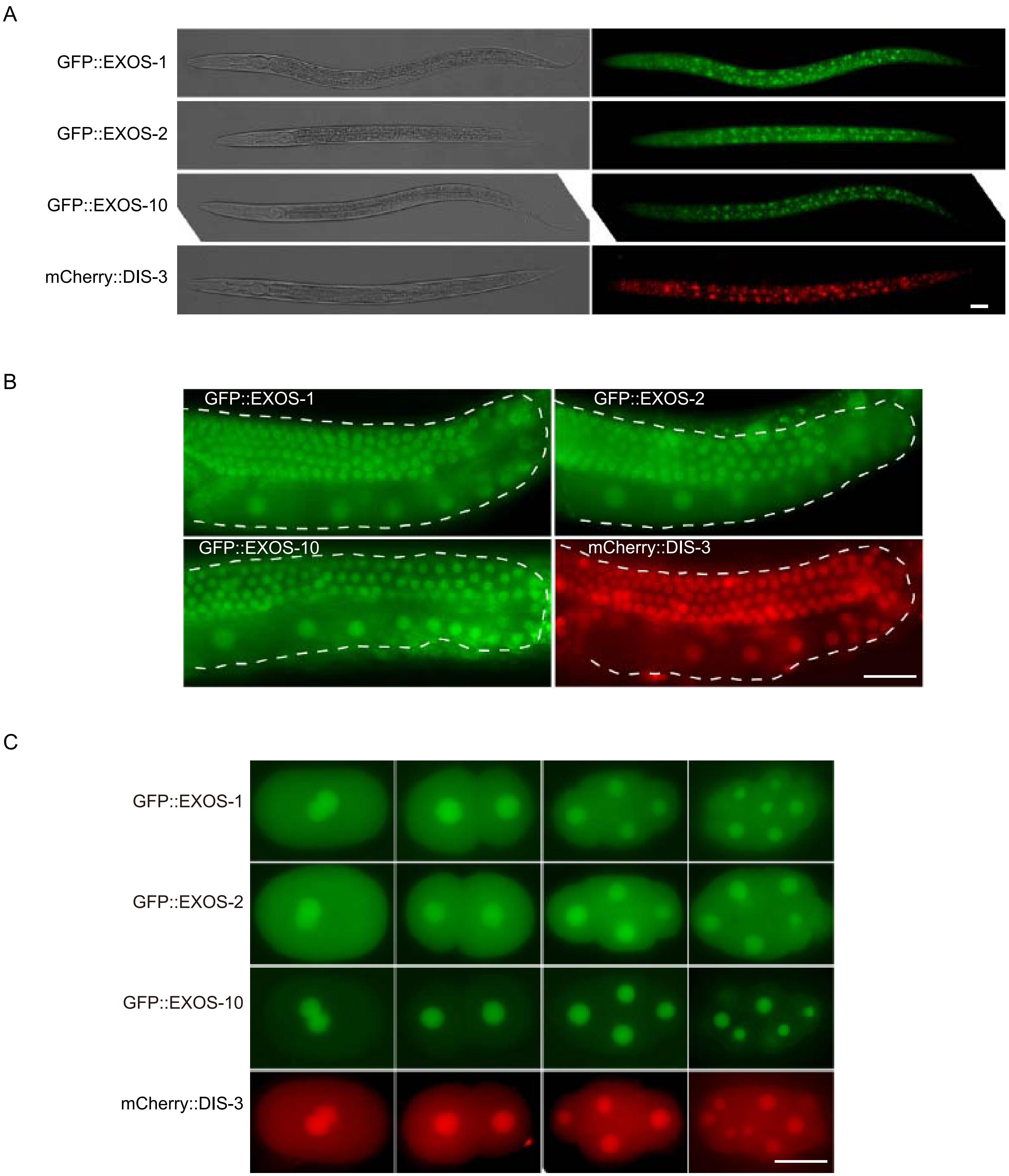
A
GFP::EXOS-1
GFP::EXOS-2
GFP::EXOS-10
mCherry::DIS-3
B
GFP::EXOS-1
GFP::EXOS-2
GFP::EXOS-10
mCherry::DIS-3
C
GFP::EXOS-1
GFP::EXOS-2
GFP::EXOS-10
mCherry::DIS-3

Figure S8

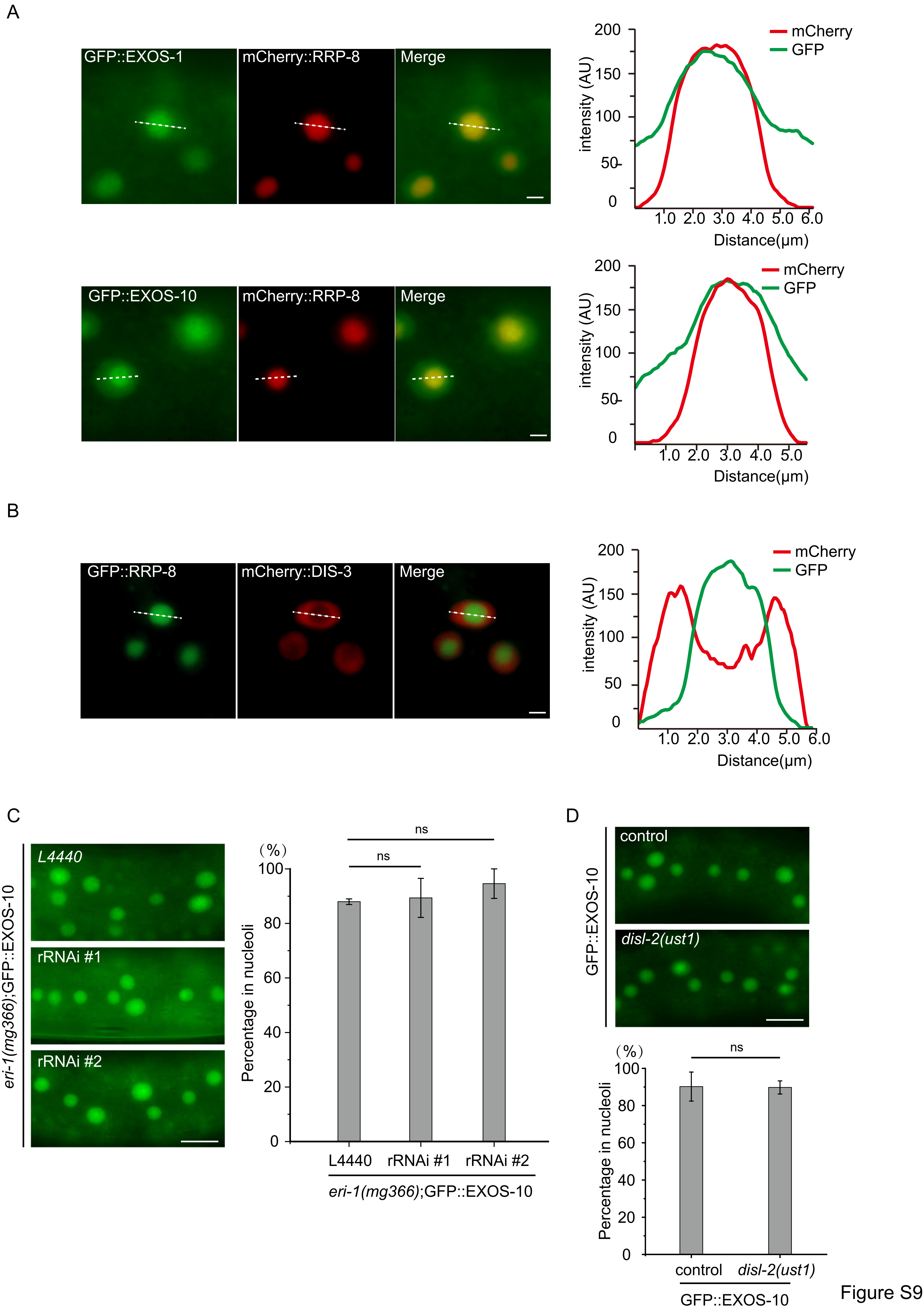
A
GFP::EXOS-1
mCherry::RRP-8
Merge
mCherry
GFP
intensity (AU)
Distance(μm)
GFP::EXOS-10
mCherry::RRP-8
Merge
B
GFP::RRP-8
mCherry::DIS-3
Merge
C
eri-1(mg366);GFP::EXOS-10
L4440
rRNAi #1
rRNAi #2
ns
(%)
Percentage in nucleoli
D
GFP::EXOS-10
control
disl-2(ust1)

Figure S9

A

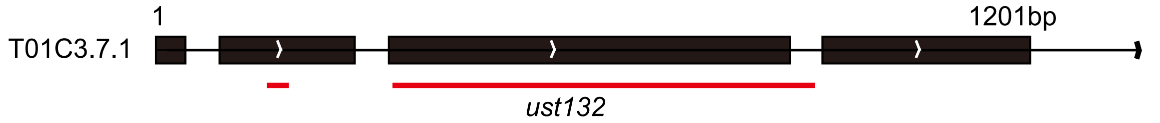

WT: CGGTGGTCGTGG(24bp)CAGTTTCG(134bp)GTGGTTTT(573bp)atatttatattacag

fib-1(ust132): CGGTGGTCGTGG------CAGTTTCG(134bp)GTGGTTTT--TAT--atatttatattacag (-597bp, +3bp)

144 917

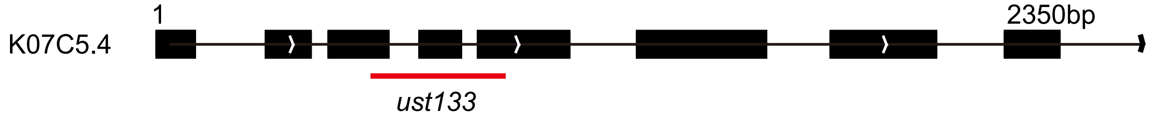

WT: TACTGAAGCGTT(339bp)GGACAAGGATATCAA

nol-56(ust133): TACTGAAGCGTT-------GGACAAGGATATCAA (-339bp)

553 918

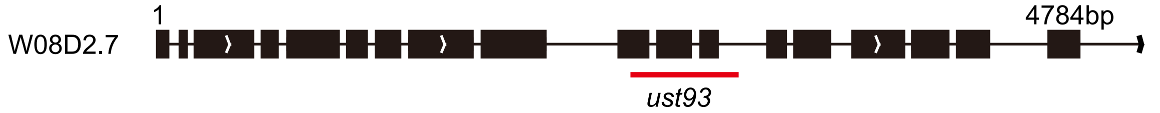

WT: AGAGTTGAAGGA(339bp)tcaaaatgtaaaaca

mtr-4(ust93): AGAGTTGAAGGA-------tcaaaatgtaaaaca (-534bp)

2460 3020

Figure S10

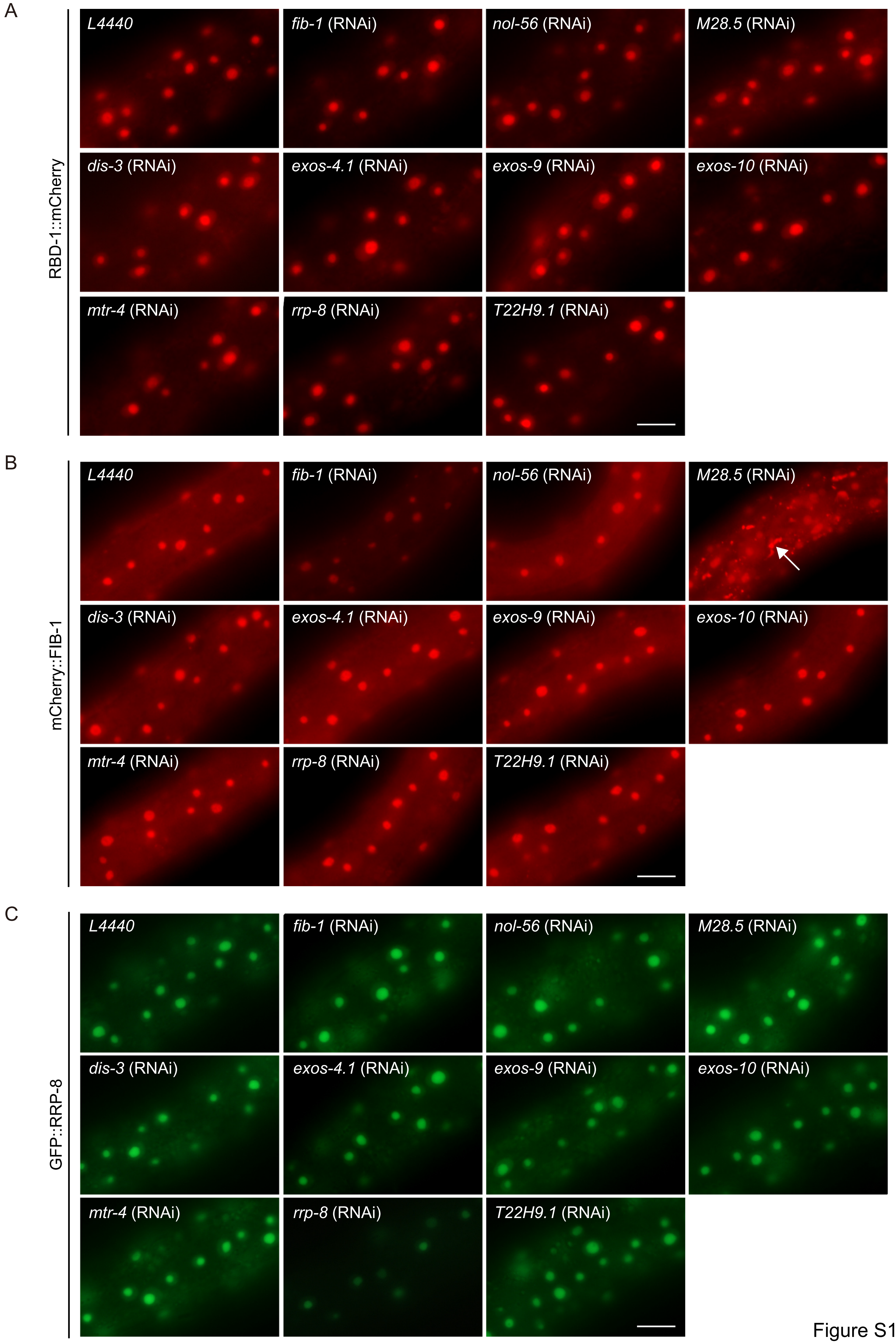
A
RBD-1::mCherry
L4440
fib-1 (RNAi)
nol-56 (RNAi)
M28.5 (RNAi)
dis-3 (RNAi)
exos-4.1 (RNAi)
exos-9 (RNAi)
exos-10 (RNAi)
mtr-4 (RNAi)
rrp-8 (RNAi)
T22H9.1 (RNAi)
B
mCherry::FIB-1
L4440
fib-1 (RNAi)
nol-56 (RNAi)
M28.5 (RNAi)
dis-3 (RNAi)
exos-4.1 (RNAi)
exos-9 (RNAi)
exos-10 (RNAi)
mtr-4 (RNAi)
rrp-8 (RNAi)
T22H9.1 (RNAi)
C
GFP::RRP-8
L4440
fib-1 (RNAi)
nol-56 (RNAi)
M28.5 (RNAi)
dis-3 (RNAi)
exos-4.1 (RNAi)
exos-9 (RNAi)
exos-10 (RNAi)
mtr-4 (RNAi)
rrp-8 (RNAi)
T22H9.1 (RNAi)

Figure S11

Table S1: Candidate-base RNAi screening for factors affecting the subcellular localization of GFP::EXOS-10.

gene ID	gene name	NCL%	yeast	human	predicted functions
L4440(control)	/	95.5%			
K07C5.4	*nol-56*	0%	NOP56	NOP56	histone methyltransferase binding activity and snoRNA binding activity
T01C3.7	*fib-1*	4.6%	FIB1	FBL	have RNA binding activity and methyltransferase activity
M28.5	*phi-9*	11.9%	SNU13	SNU13	box C/D snoRNP complex
W08D2.7	*mtr-4*	22.7%	MTR4	MTR4	have ATP binding activity, RNA binding activity, and RNA helicase activity
F10B5.1	*rpl-10*	31.8%	RPL10	RPL10	ribosomal large subunit assembly
F37C12.13	*exos-9*	60.0%	RRP45	EXOSC9	exonucleolytic trimming to generate mature 3'-end of 5.8S rRNA from tricistronic rRNA transcript
C04G2.6	*dis-3*	68.2%	RRP44	DIS3	exosome endoribonuclease and 3'-5' exoribonuclease
ZK265.6	*nol-16*	75.0%	NOP16	NOP16	ribosomal large subunit biogenesis
K09B11.2	*nol-9*	78.8%	GRC3	NOL9	Polynucleotide 5'-kinase involved in rRNA processing
F25H8.2	/	85.0%	NAF1	NAF1	box H/ACA snoRNP assembly
K01G5.5	/	90.9%	CBF5	DKC1	box H/ACA snoRNP complex
F32E10.1	*nol-10*	91.7%	NOL10	NOL10	an ortholog of human NOL10 (nucleolar protein 10); exhibits RNA binding activity
C27H6.2	*ruvb-1*	95.0%	RUVB1	RUVBL1	involved in TOR signaling, box C/D snoRNP assembly
Y48A6B.3	/	95.0%	NHP2	NHP2	box H/ACA snoRNP complex
Y66H1A.4	/	95.5%	GAR1	GAR1	box H/ACA snoRNP complex

Table S2: Strains used in the work.

Genotype
N2
CB4856
dis-3(ust56)
exos-1(ust57)
eri-1(mg366);3XFLAG::GFP::NRDE-3(ggIS1)
eri-1(mg366);dis-3(ust56);3XFLAG::GFP::NRDE-3(ggIS1)
eri-1(mg366);dis-3(ok357);3XFLAG::GFP::NRDE-3(ggIS1)
eri-1(mg366);dis-3(ust56);mCherry::DIS-3(ustIS115);3XFLAG::GFP::NRDE-3(ggIS1)
eri-1(mg366);dis-3(ust56);rrf-1(pk1417);3XFLAG::GFP::NRDE-3(ggIS1)
eri-1(mg366);dis-3(ust56);rrf-2(ok210);3XFLAG::GFP::NRDE-3(ggIS1)
eri-1(mg366);dis-3(ust56);rrf-3(pk1426);3XFLAG::GFP::NRDE-3(ggIS1)
eri-1(mg366);dis-3(ust56);rrf-1(pk1417);rrf-2(ok210);3XFLAG::GFP::NRDE-3(ggIS1)
eri-1(mg366);exos-1(ust57);3XFLAG::GFP::NRDE-3(ggIS1)
eri-1(mg366);exos-2(tm6653);3XFLAG::GFP::NRDE-3(ggIS1)
eri-1(mg366);exos-3(tm6844);3XFLAG::GFP::NRDE-3(ggIS1)
eri-1(mg366);exos-4.1(tm5568);3XFLAG::GFP::NRDE-3(ggIS1)
eri-1(mg366);exos-5(ust61);3XFLAG::GFP::NRDE-3(ggIS1)
eri-1(mg366);exos-7(ust62);3XFLAG::GFP::NRDE-3(ggIS1)
eri-1(mg366);exos-8(ust60);3XFLAG::GFP::NRDE-3(ggIS1)
eri-1(mg366);exos-9(ok1635);3XFLAG::GFP::NRDE-3(ggIS1)
eri-1(mg366);exos-10(ok2269);3XFLAG::GFP::NRDE-3(ggIS1)
3XFLAG::GFP::EXOS-1(ustIS112)
3XFLAG::GFP::EXOS-2(ustIS113)
3XFLAG::GFP::EXOS-10(ustIS114)
mCherry::DIS-3(ustIS115)
mCherry::RRP-8(ustIS75)
GFP::RRP-8(ustIS76)
RBD-1::mCherry(ustIS207)

mCherry::FIB-1(ustIS36)

mCherry::DIS-3(ustIS115);GFP::RRP-8(ustIS76)

dis-3(ust56);3XFLAG::GFP::EXOS-1(ustIS112)

dis-3(ust56);3XFLAG::GFP::EXOS-10(ustIS114)

3XFLAG::GFP::EXOS-1(ustIS112);mCherry::RRP-8(ustIS75)

3XFLAG::GFP::EXOS-10(ustIS114);mCherry::RRP-8(ustIS75)

mCherry::DIS-3(ustIS115);GFP::RRP-8(ustIS76)

eri-1(mg366);fib-1(ust132);3XFLAG::GFP::NRDE-3(ggIS1)

eri-1(mg366);nol-56(ust133);3XFLAG::GFP::NRDE-3(ggIS1)

eri-1(mg366);mtr-4(ust93);3XFLAG::GFP::NRDE-3(ggIS1)

3XFLAG::GFP::RPOA-2(ustIS116)

3XFLAG::GFP::RPOA-2(ustIS116);mCherry::FIB-1(ustIS36)

GFP::NRDE-2(ustIS117);mCherry::RPOA-2(ustIS116)

eri-1(mg366);3XFLAG::GFP::RPOA-2(ustIS116)

eri-1(mg366);nrde-2(gg91);3XFLAG::GFP::RPOA-2(ustIS116)

Table S3. Sequences of sgRNAs for CRISPR/Cas9-mediated gene editing.

rpoa-2_sg#1	TTCAGTTCGGCCACAATTCG
rpoa-2_sg#2	ATTGTGGCCGAACTGAACAG
rpoa-2_sg#3	GCGACAGCCACTGTTCAGTT
exos-1_sg#1	ACAAGGTTCTCGACGCGAT
exos-1_sg#2	ATCACTTGCACCAGGTTGT
exos-1_sg#3	ATACTGATGATGTCACATT
exos-1_sg#4	CCGACAGCCATCACTTTGG
exos-5_sgRNA #1	GAGTGATGAGGCTATGACTC
exos-5_sgRNA #2	GTACATGGAATTCAGGATGA
exos-5_sgRNA #3	GCATCCAGAAGTGTGTGCGA
exos-7_sgRNA #1	GGCGAATTGACTGCTCGCGT
exos-7_sgRNA #2	GGTGACGTTGCACCAGATGA
exos-7_sgRNA #3	GTTGAGATTGTCAGCAGCAG
exos-8_sg#1	GAGTCTATCCTGACGGACG
exos-8_sg#2	GTTAGTGATGCACCCCTGG
exos-8_sg#3	TGTGGCAAACCTTTGCCTC
exos-8_sg#4	CGTCAATCGATGCATTGTT
nrde-2_sgRNA#1	GGAACAATGTTTCGAGCGTATGG
nrde-2_sgRNA#2	GAAACATTGTTCATTAAGTTTGG
fib-1_sgRNA#1	GCGGTGGTCGTGGAGGATA
fib-1_sgRNA#2	TGTCCGTCGATGACGGAGC
fib-1_sgRNA#3	CCACTTGAGCAGGTAACCC
nol-56_sgRNA#1	TCGATGCCGCTCATGCTGA
nol-56_sgRNA#2	ATCTGAAGCTTGTCTTCGG
nol-56_sgRNA#3	ATTGCCCTTCTCGATCAGT
mtr-4_sgRNA#1	TGAAGGAATGGCTGTTTCA
mtr-4_sgRNA#2	GTACAAAGTACATTGCATT
mtr-4_sgRNA#3	TCGTATATCAATGGGTTAA
mtr-4_sgRNA#4	GCCAAGGCTTTAGCGAATA

Table S4. Sequences of quantitative real-time PCR primers for ChIP experiments.

eft-3 qRT F	CAAGGATATTCGTCGTGGATCC
eft-3 qRT R	AATCGAGAACTGGAGTGTATCCG
ama-1 qRT F	CGAACCTGCCGATTGATA
ama-1 qRT R	ACCACGATTGACCAACTC
lin-15b qRT #1F	ACCGAGACCAGCCAATGT
lin-15b qRT #1R	TCTTCATCCAGTGGTTCATCCT
lin-15b qRT #2F	CACTGAACTCACAAGACCACAC
lin-15b qRT #2R	TCCAATTTGAAGTCATCCCTCTG
5ETS-1F	CCTACACTCATGTCTTTGCAGA
5ETS-1R	GCCGTACTATGCAGCAAGG
5ETS-2F	CCACATTCAGAGGCTGGTGA
5ETS-2R	CCTCTCACCAGCCTATCATTCG
5ETS-3F	CGTCTCTCAAATTGCACACTGC
5ETS-3R	CCTGAGACATCACGTCTCAGAC
rDNA#1 F	CAAGACCAATACCGCAACATCA
rDNA#1 R	TCATTGCGCCGATCCATAGAT
rDNA#2 F	GGAGCTAATACATGCAACTATACCC
rDNA#2 R	CAGTCGAAACTGACAGTAAACTGC
rDNA#3 F	GGATGAGTTATTTCAATGAGTTGAATAC
rDNA#3 R	CGCAGCAATAACGAGATACACTAG
rDNA#4 F	CTTCGAGTAGCAAGGAGAGG
rDNA#4 R	GCACGTAACCTAGATCCAACTAC
rDNA#5 F	GGACTGTCGCTTCGAGGTTTAA
rDNA#5 R	GCGATGATCCAGCTGCAG
rDNA#6 F	GAATCAGTACACTGATTGCCAAAAG
rDNA#6 R	GTTCTTCATCGATACTCGATGC
rDNA#7 F	CGATAGCGAACAAGTACC
rDNA#7 R	TCTCCAAGCAACATCAAC
rDNA#8 F	TAATGTCCTCAACCTATTCTCAA
rDNA#8 R	GCCAGTTCTGCTTACCAA
rDNA#9 F	GGAATCCGACTGTCTAAT
rDNA#9 R	CGCTTACTCGAATTACTAC
rDNA#10 F	CATACGACTTGGTCTCTTGG
rDNA#10 R	CTGCAAAGACATGAGTGTAGG
rDNA#11 F	GTCTGGTTTATTCCGATAACGAG
rDNA#11 R	GACCTGTTATCGCTCAATCTC
rDNA#12 F	CTCGGCTGATCATCAAGACG
rDNA#12R	CGATATCAACACACACACGAGAC
rDNA#13F	GTGAACTGTCAACGTGAATGC
rDNA#13R	GGTCTCATGAGCGAGAAGTTAG

Table S5. Sequences of quantitative real-time PCR primers for detecting the pre-rRNA and rRNA levels.

eft-3 E2L F	GTAAGGGATCTTTCAAGTACGC
eft-3 E2L R	CATCGATGATGGTGATGTAGTAC
5S 1F	CTCTCCACACAACACACAGC
5S 1R	ATATGGTCGTAAGCGTCTATGG
lin-15b pre#1 F	CAATTCCTAACAATCATAAACTCTCAA
lin-15b pre#1 R	GGAGGTATATAAGGTGGTTGAATGTTTC
lin-15b pre#2 F	CAATTCCTAACAATCATAAACTCTCAA
lin-15b pre#2 R	CATTGTCAATGTAGTGGAGGATG
pre#1 F	TCATTGCGCCGATCCATAGAT
pre#1 R	CAAGACCAATACCGCAACATCA
pre#2 F	CAAGACCAATACCGCAACATCA
pre#2 R	CGCAGACATATAGTCTAGCGAG
pre#3 F	CCCTTGCTGCATAGTACGGC
pre#3 R	TCAGCAGCATGTCAATGTGGT
pre#4 F	GGACACACCACCAAAGTCTCAA
pre#4 R	TTGAGAGACGGCAGACAACG
pre#5 F	CTGTATGAGTGTCCCATCTCACG
pre#5 R	GCATGGCTTAATCTTTTACTTGAGC
pre#6 F	CGCATTGGTTTGAACCGG
pre#6 R	CAACTGACCGTGAAGCCAG
pre#7 F	CGTAACAAGGTAGCTGTAGGTG
pre#7 R	CTGGAACGTTGACATTTCGAC
pre#8 F	CATACGACTTGGTCTCTTGG
pre#8 R	CTGCAAAGACATGAGTGTAGG
18S#1 F	CGATAACAGGTCTGTGATGCCC
18S#1 R	TACCCTATCCCGGACATGGAAG
26S#1 F	TAATGTCCTCAACCTATTCTCAA
26S#1 R	GCCAGTTCTGCTTACCAA
26S#2 F	GGAATCCGACTGTCTAAT
26S#2 R	CGCTTACTCGAATTACTAC
